# Prenatal Androgen Treatment Does Not Alter the Firing Activity of Hypothalamic Arcuate Kisspeptin Neurons in Female Mice

**DOI:** 10.1523/ENEURO.0306-21.2021

**Published:** 2021-09-29

**Authors:** Amanda G. Gibson, Jennifer Jaime, Laura L. Burger, Suzanne M. Moenter

**Affiliations:** 1 Departments of Molecular and Integrative Physiology, University of Michigan, Ann Arbor, MI 48109; 2 Neuroscience Graduate Program, University of Michigan, Ann Arbor, MI 48109; 3 Internal Medicine, University of Michigan, Ann Arbor, MI 48109; 4 Obstetrics and Gynecology, University of Michigan, Ann Arbor, MI 48109; 5 The Reproductive Sciences Program, University of Michigan, Ann Arbor, MI 48109

**Keywords:** arcuate, development, kisspeptin, reproduction

## Abstract

Neuroendocrine control of reproduction is disrupted in many individuals with polycystic ovary syndrome (PCOS), who present with increased luteinizing hormone (LH), and presumably gonadotropin-releasing hormone (GnRH), release frequency, and high androgen levels. Prenatal androgenization (PNA) recapitulates these phenotypes in primates and rodents. Female offspring of mice injected with dihydrotestosterone (DHT) on gestational days 16–18 exhibit disrupted estrous cyclicity, increased LH and testosterone, and increased GnRH neuron firing rate as adults. PNA also alters the developmental trajectory of GnRH neuron firing rates, markedly blunting the prepubertal peak in firing that occurs in three-week (3wk)-old controls. GnRH neurons do not express detectable androgen receptors and are thus probably not the direct target of DHT. Rather, PNA likely alters GnRH neuronal activity by modulating upstream neurons, such as hypothalamic arcuate neurons co-expressing kisspeptin, neurokinin B (gene Tac2), and dynorphin, also known as KNDy neurons. We hypothesized PNA treatment changes firing rates of KNDy neurons in a similar age-dependent manner as GnRH neurons. We conducted targeted extracellular recordings (0.5–2 h) of Tac2-identified KNDy neurons from control and PNA mice at 3wks of age and in adulthood. About half of neurons were quiescent (<0.005 Hz). Long-term firing rates of active cells varied, suggestive of episodic activity, but were not different among groups. Short-term burst firing was also similar. We thus reject the hypothesis that PNA alters the firing rate of KNDy neurons. This does not preclude altered neurosecretory output of KNDy neurons, involvement of other neuronal populations, or *in vivo* networks as critical drivers of altered GnRH firing rates in PNA mice.

## Significance Statement

Prenatal androgenization (PNA) recapitulates key aspects of the common reproductive disorder polycystic ovary syndrome (PCOS). It is postulated that disruptions in the episodic pattern of gonadotropin-releasing hormone (GnRH) secretion in part underly this disorder, yet GnRH neurons do not express androgen receptor to respond directly to elevated androgens. A population of kisspeptin, neurokinin B, and dynorphin-expressing (KNDy) neurons in the hypothalamic arcuate nucleus (ARC) are thought to regulate pulsatile GnRH release and some express androgen receptor. We did not find evidence, however, that PNA altered spontaneous activity of KNDy neurons before puberty at three weeks (3wks) of age or in adulthood. This suggests that PNA likely acts via other components of the broader hypothalamic network to change the patterns of GnRH release.

## Introduction

Gonadotropin-releasing hormone (GnRH) regulates the secretion of the gonadotropins luteinizing hormone (LH) and follicle-stimulating hormone (FSH) from the anterior pituitary. GnRH is released in an episodic manner that varies in frequency through the female reproductive cycle (Levine and Ramirez, 1982; Moenter et al., 1991). Lower frequency favors synthesis and release of FSH over LH and is important for recruiting ovarian follicles and their subsequent maturation, whereas higher pulse frequency in the mid-late follicular phase favors LH, which drives androgen synthesis (Wildt et al., 1981; Haisenleder et al., 1991). Failure to vary the frequency of GnRH release is thought to be a key neuroendocrine phenotype of the reproductive disorder polycystic ovary syndrome (PCOS).

PCOS is a complex spectrum of reproductive and metabolic phenotypes with postulated genetic and environmental causes. Patients with PCOS often exhibit a persistently high LH (and presumably GnRH) pulse frequency, leading to disrupted follicle maturation and ovulation. Increased LH stimulation also drives hyperandrogenism (McCartney et al., 2002; Dumesic et al., 2015). To investigate the etiology of the disorder, animal models are needed for experimental manipulations and measurements that cannot be conducted in humans. Elevated prenatal androgen (PNA) exposure recapitulates PCOS-like reproductive phenotypes in many species including non-human primates (Dumesic et al., 1997; Abbott et al., 2008, 2017, 2019), sheep (Birch et al., 2003; Veiga-Lopez et al., 2008), and rodents (Sullivan and Moenter, 2004; Foecking et al., 2005). In adult-PNA mice, LH pulse frequency (Moore et al., 2015) and GnRH neuron action potential firing frequency (Roland and Moenter, 2011) are both increased. PNA in mice also alters the developmental trajectory of GnRH neuron firing frequency, which is interesting as aspects of PCOS may emerge around the pubertal transition (McCartney et al., 2002; Rosenfield, 2007). Specifically, in control mice, the firing frequency peaks at 3wks of age before decreasing to adult levels (Dulka and Moenter, 2017). In contrast, the firing frequency in PNA female mice did not vary with age, and it was lower than control mice at 3wks, distinct from the increase observed in PNA adults (Sullivan and Moenter, 2004; Dulka and Moenter, 2017)

The mechanisms by which PNA alters the activity of GnRH neurons are not completely understood. These neurons do not express detectable levels of androgen receptor (Herbison et al., 1996), thus it is likely that upstream neuronal populations are involved in regulating their firing patterns. One such population is in the hypothalamic arcuate nucleus (ARC), specifically neurons that co-express kisspeptin, neurokinin B, and dynorphin (KNDy neurons). KNDy neurons are posited to be involved in the control of pulsatile GnRH, and subsequent LH, secretion (Han et al., 2015; Clarkson et al., 2017; McQuillan et al., 2019). KNDy neurons express receptors for gonadal steroids, including androgen receptor (Smith et al., 2005), and could serve as the site of steroidal feedback that alters GnRH neuron activity and/or a site of action for PNA exposure (Oakley et al., 2009; Caldwell et al., 2017; Vanacker et al., 2017; Walters et al., 2018).

We hypothesized that PNA treatment would alter the firing frequency of KNDy neurons in an age-dependent manner similar to that of GnRH neurons. We tested this by assessing the effect of PNA on the spontaneous firing frequency of KNDy neurons in prepubertal three-week (3wk)-old and adult female mice through long-term extracellular recordings. Specifically, we postulated that PNA treatment would increase KNDy neuron activity relative to controls in adults but reduce activity relative to controls in 3wk-old mice. We also predicted that 3wk-old control mice would exhibit increased KNDy neuron firing frequency relative to control adults and 3wk-old PNA mice.

## Materials and Methods

### Animals

Mice expressing enhanced green fluorescent protein (GFP) under the control of Tac2 promoter (Tac2-GFP), BAC transgenic mice (015495-UCD/STOCK Tg [Tac2-EGFP]381Gsat, Mouse Mutant Regional Resource Center; http://www.mmrrc.org/) were used to identify KNDy neurons for recording. In mice, *Tac2* encodes neurokinin B, which is co-expressed with kisspeptin and dynorphin in KNDy neurons. Tac2-GFP-identified cells in brain slices used for recording also express kisspeptin and/or dynorphin at high percentages, supporting their identity as KNDy neurons (Ruka et al., 2013). Mice were maintained in a 14/10 h light/dark photoperiod (lights on at 3 A.M. Eastern Standard Time) and had *ad libitum* access to water and either Harlan 2919 chow during pregnancy/lactation or 2916 chow for maintenance. All animal procedures were approved by the University of Michigan Institutional Animal Care and Use Committee.

To generate experimental mice, a Tac2-GFP female and a CD1 female mouse were bred with a C57B/6 male and monitored daily for a copulatory plug (day 1 of pregnancy). The CD1 dam assists in providing maternal care and nutrition. On days 16–18 of pregnancy, dams were injected subcutaneously with 225 μg/d of dihydrotestosterone (DHT) or sesame oil as vehicle. Control offspring from dams for whom timing of pregnancy could not be clearly established were also included in studies without injections; firing rate from these mice did not differ from vehicle-treated mice (treatment: *F*_(1,22)_ = 2.114, *p *= 0.160; interaction of age and treatment: *F*_(1,22)_ = 0.237, *p* = 0.631). Experiments were conducted on female offspring before weaning at 3wks of age [postnatal day (PND) 18–PND 21] or in adulthood (PND 66–PND 152; median 133). PNA status was confirmed by anogenital distance and estrous cyclicity in adults. Anogenital distance was measured with digital calipers on two to three successive days and averaged for each mouse. Estrous cyclicity was assessed via vaginal cytology and studies on adult females were done on diestrus. Cycle stage was confirmed with uterine mass; one DHT-treated mouse was excluded because of a uterine mass of 136.2 mg, suggestive of incorrect cycle identification based on vaginal cytology.

### Brain slice preparation

All solutions were bubbled with 95% O_2_/5% CO_2_ for at least 15 min before tissue exposure and throughout the procedures. The brain was rapidly removed and cooled for 60 s in ice-cold sucrose saline solution containing the following: 250 mm sucrose, 3.5 mm KCl, 26 mm NaHCO_3_, 10 mm D-glucose, 1.25 mm Na_2_HPO_4_, 1.2 mm MgSO_4_, and 3.8 mm MgCl_2_. Coronal slices (300 μm) through the hypothalamic region, including the ARC, were cut with a Leica VT1200S (Leica Biosystems). Slices were incubated for 30 min at room temperature in 50% sucrose saline and 50% artificial CSF (ACSF) containing the following: 135 mm NaCl, 3.5 mm KCl, 26 mm NaHCO_3_, 10 mm D-glucose, 1.25 mm Na_2_HPO_4_, 1.2 mm MgSO_4_, and 2.5 mm CaCl_2_ (pH 7.4). The slices were then held in 100% ACSF at room temperature for between 0.5 and 5.5 h before recording. No differences in results were attributable to duration after brain slice preparation.

### Electrophysiological recording

To evaluate the long-term firing patterns of KNDy neurons with minimal disruption of the cell’s intrinsic properties, targeted single-unit extracellular recordings were conducted (Nunemaker et al., 2003; Alcami et al., 2012). Individual slices were transferred to a recording chamber mounted on the stage of an Olympus BX51WI upright fluorescent microscope. A constant perfusion of ACSF at a rate of 3 ml/min was established with a MINIPULS 3 peristaltic pump (Gilson). The chamber was maintained at a temperature of 29–32°C with an inline heating system (Warner Instrument Corporation). ACSF was replaced every hour.

Recording electrodes (resistance 2–4 MΩ) were pulled from borosilicate glass (Schott no. 8250; World Precision Instruments) using a Sutter P-97 puller (Sutter Instrument). The pipettes were filled with a HEPES-buffered pipette solution containing the following: 150 mm NaCl, 10 mm HEPES, 10 mm D-glucose, 2.5 mm CaCl_2_, 1.3 mm MgCl_2_, and 3.5 mm KCl, pH7.4. At the surface of the brain slice, a small amount of negative pressure was applied to bring the pipette in contact with tissue, facilitating the later formation of a low-resistance seal (<100 MΩ) between the pipette and neuron (Alcami et al., 2012). Recordings were made with one channel of an EPC10 dual patch-clamp amplifier using PatchMaster software (HEKA Elektronic). Cells were held in voltage-clamp with a 0-mV pipette holding potential. Seal resistance was checked every 10–15 min by measuring response to a 5-mV hyperpolarizing step between series. Data were acquired at 10 kHz and filtered at 5 kHz.

Recording duration ranged from 0.5 to 2.6 h (mean ± SEM 71.7 ± 2.9 min; median 60 min). If a cell was not firing at the conclusion of a recording session, either 20 mm-potassium ACSF (two cells) or the neurokinin-3 receptor agonist senktide (100 nm; Phoenix Pharmaceuticals, 42 cells) was bath-applied. If a cell failed to exhibit action currents following either stimulus, recording integrity could not be verified and data analysis was truncated to the last action current, or not included in analyses if there were no spontaneous events. Response to senktide was quantified by comparing the spontaneous firing frequency for the 5 min before addition of senktide to the ACSF to the firing frequency for 5 min, beginning 2 min after senktide reached the bath to allow time for the drug to equilibrate in the chamber and penetrate the slice. This 2-min delay was chosen based on the onset of and peak senktide response across cells.

### Analysis

Event detection was completed using IgorPro8 (WaveMetrics) using custom routines, and all events were manually confirmed. The average spontaneous firing rate was calculated for each cell as total events/recording duration. The short-term patterns of neuronal activity were also assessed with custom IgorPro8 routines. Repetitive, grouped firing events are referred to as “bursts” for analysis. To be considered part of a burst, a firing event must occur within a defined “burst window” after the previous event. The burst window for analysis of these KNDy neurons was identified by varying the burst window from 0.01 to 1 s in 10-ms intervals and selecting the burst window that captures the maximal burst frequency for the control cells; this was 230 ms as in prior reports (Vanacker et al., 2017). At the selected burst window, the software characterizes each event as belonging to a burst or as a single spike, then calculates the following parameters: burst frequency, burst duration, intraburst interval, spikes per burst, single spike frequency, and interevent interval. Burst duration and spikes per burst are the averages for all bursts from a given cell. Intraburst interval is the average of intervals between spikes in a burst, whereas interevent interval is the average of intervals greater than the burst window and can occur between bursts, between single spikes or between single spikes and bursts. Short breaks in the recording (typically <2 s) occur at 10- to 15-min intervals to monitor the seal resistance. Intervals that crossed these gaps were not included when calculating cells’ averages. Spikes that occurred within 230 ms (i.e., the burst window) of these gaps, or the start or end of the recording, were characterized according to the available information; this could lead to an underestimate of the burst frequency, burst duration, and/or spikes per burst.

### PCR to assess arcuate gene expression

Hypothalamic tissue punches were collected to assess the effect of PNA on gene expression. Separate cohorts of mice from those used for recordings were used to collect tissue micro-punches from the ARC. A coronal slice was obtained with an adult mouse brain matrix (1 mm, Zivic Instruments); an initial cut was made just caudal to the optic chiasm, followed by a cut just rostral to the brain stem (2–3 mm thick) for the ARC. Tissue punches were made with a 1.2-mm Palkovits punch. Tissue was immediately homogenized in RLT buffer (QIAGEN) containing 2-mercaptoethanol (1%v/v, Sigma), snap frozen, and stored at −80°C. RNA from was extracted with the RNeasy Micro kit with on-column DNasing (QIAGEN); 240-ng RNA per sample was reverse transcribed with Superscript IV VILO Master Mix (Fisher/Invitrogen). A standard curve of hypothalamic RNA (600, 120, 24, 4.8, and 0 ng/20 μl) was also reverse transcribed (Ruka et al., 2013). The transcripts for: *Kiss1*, *Kiss1r*, *Pdyn*, *Oprk1*, *Tac2*, *Tacr3*, *Ar*, *Esr1*, and *Pgr* were assayed via Taqman quantitative PCR in duplicate with 10-ng cDNA. Data were analyzed by the ΔΔCT method (Bustin, 2002), normalized to *Actb* and *Syn1* and reported relative to 3wk-CON. Primers and Taqman probes were purchased from Integrated DNA Technologies and are reported in Table 1.

**Table 1:** Probes and primers sequences for PCR experiments (**Fig. 5**)

Transcript	Probe 5′—>3′	Forward 5′—>3′	Reverse 5′—>3′
*Actb*	CTG GCC TCA CTG TCC ACC TTC C	GAT TAC TGC TCT GGC TCC TAG	GAC TCA TCG TAC TCC TGC TTG
*Syn1*	ACG TGT CTA CCC ACA ACT TGT ACC TG	CTT GAG CAG ATT GCC ATG TC	ACC TCA ATA ATG TGA TCC CTT CC
*Kiss1*	CGG ACT GCT GGC CTG TGG AT	CTG CTT CTC CTC TGT GTC G	TTC CCA GGC ATT AAC GAG TTC
*Kiss1r*	TCA ATC CGC TGC TCT ATG CCT TCC	CTC ACT GCA TGT CCT ACA GC	GCC TGT CTG AAG TGT GAA CC
*Tac2*	AGC TTT GTC CTT CAG GCA CCA TGA	CTG CAC TCT TGT CTC TGT CT	ACA GCC GCA AAC AGC AT
*Tacr3*	TCT CTT GAA GCC TGC ACG AAA TCT TTT G	AGC TCA ACC ATG TAC AAC CC	CTC ATC GTA GCT GGA GAC TTG
*Pdyn*	TCA ACC CCC TGA TTT GCT CCC TG	GTG CAG TGA GGA TTC AGG ATG	CAT GTC TCC CAC TCC TCT GA
*Opkr1*	AGA GAA TTG CCC ACT AAG CCC ACC	CAT CAC CGC TGT CTA CTC TG	GGT CTT CAT CTT CGT GTA TCG G
*Ar*	ACC ACA TGC ACA AGC TGC CTC T	CTG CCT TGT TAT CTA GCC TCA	ATA CTG AAT GAC CGC CAT CTG
*Esr1*	TGC CTT CCA CAC ATT TAC CTT GAT TCC T	CCT GTT TGC TCC TAA CTT GCT	GAA CCG ACT TGA CGT AGC C
*Pgr*	AGA TTC AGA AGC CAG CCA GAG CC	CGC CAT ACC TTA ACT ACC TGA G	CCA TAG TGA CAG CCA GAT GC

### Statistics

Data visualization and analyses were conducted with R (R Core Team, 2019) and RStudio (RStudio Team, 2019) using a combination of open-sourced packages (Chang, 2014; Xie, 2014, 2015, 2020; Fox and Weisberg, 2019; Wickham et al., 2019, 2020; Chang et al., 2020; Gohel, 2020a,b; Henry and Wickham, 2020a,b; Kassambara, 2020, 2021; Schauberger and Walker, 2020; Schloerke et al., 2020; Wickham and Hester, 2020; Wickham and Seidel, 2020; Wilke, 2020; Zhu, 2020; Xie et al., 2021) and custom procedures. Additional statistical analyses were conducted with Prism 9 (GraphPad). Data are reported as mean ± SEM, with median illustrated where indicated. For recordings, n is number of cells; for PNA phenotype confirmation and mRNA quantification, n is number of mice. Normality of the data distribution was evaluated with Shapiro–Wilk. Two-way ANOVA (Type III) was conducted to evaluate the main effects and interactions of age and prenatal treatment. Bonferroni correction for multiple comparisons was used as this test is sufficiently robust for non-normally distributed data (Underwood, 1997). The level accepted as significant was set to *p *<* *0.05. Statistical tables for two-way ANOVAs report the differences in means and associated 95% confidence interval (CI) defined for age (adult – 3wk), treatment (PNA – control), and interaction ([adult-PNA – adult-CON] – [3wk-PNA – 3wk-CON]).

### Software accessibility

The event detection and burst analysis code described in the paper is freely available online at https://gitlab.com/um-mip/coding-project. The R analysis code is freely available online at https://github.com/gibson-amandag/PNA_KNDy. The code for IgorPro and R analysis are also provided as Extended Data 1. Analyses were conducted on a MacBook Pro, Early 2015 version, running macOS Catalina 10.15.7 and on a Mac Mini, 2018 version, running macOS Mojave 10.14.6

10.1523/ENEURO.0306-21.2021.ed1 Extended Data 1 Code used to detect and analyze events in Igor Pro (coding-project) and for data analysis in R (PNA-KNDy). Download Extended Data 1, ZIP file.

## Results

### PNA characterization

**Figure 1.**
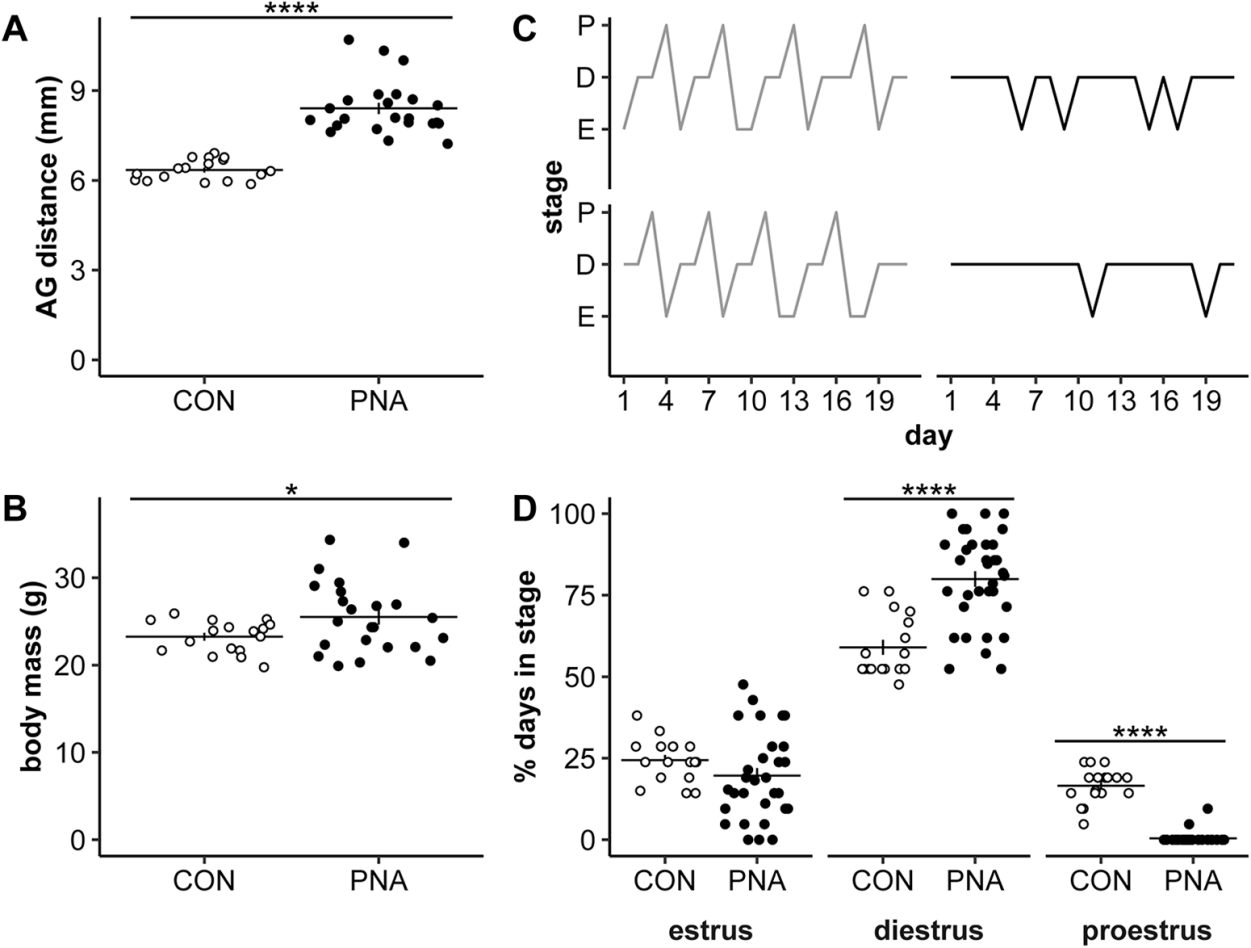
Confirmation of PNA phenotype in adults and surviving littermates of 3wk-old mice used for electrophysiology. ***A***, ***B***, ***D***, Individual values (CON open symbols, PNA black symbols) and mean ± SEM for anogenital (AG) distance (***A***), body mass (***B***), or percent days in each cycle stage (***D***). ***C***, Representative estrous cycles over a 3wk period; P, proestrus; D, diestrus; E, estrus. CON are shown on the left in grey, and PNA are on the right in black. Statistical parameters reported in Table 2; *p < 0.05, ****p ≤ 0.0001.

To verify the effects of PNA (225 μg/d of DHT administered to dams on gestational days 16–18), anogenital distance, body mass, and estrous cycles were recorded from adult offspring, including the surviving female littermates of 3wk-old mice that were used for recordings where possible. As adults, PNA mice had a longer anogenital distance (Fig. 1*A*; statistical parameters in Table 2; control *n* = 17 mice from 10 litters, PNA *n* = 23 mice from 11 litters, *p *<* *0.0001) and larger body mass (Fig. 1*B*, *p* = 0.026) than control mice. PNA treatment also altered the distribution of days spent in each estrous cycle stage (Fig. 1*C*,*D*, *p* < 0.0001). PNA mice spent more days in diestrus than expected (standardized residual = 7.08) and fewer days in proestrus than expected (standardized residual = −10.11). These results indicate that the PNA treatment was successful.

**Table 2:** Statistical parameters characterizing the PNA phenotype (**Fig. 1**)

Property	Two-tailed unpaired Welch- corrected Student’s *t* test	Mean difference	Effect size, Cohen’s *d*		
Anogenital distance (mm)	*t*_(30.13)_ = 10.052, ***p *<* *0.0001**	Diff 2.06 [CI 1.64, 2.48]	*d *=* *3.03		
Body mass (g)	*t*_(32.03)_ = 2.331, ***p *=* *0.0262**	Diff 2.26 [CI 0.29, 4.24]	*d *=* *0.707		
Property	χ^2^ test		Estrus	Diestrus	Proestrus
Estrous cycle stage distribution	χ^2^ = 111.392, *n* =* *992, df* *=* *2, ***p *<* *0.0001**	Std resid:	−1.79	7.08	10.11
		Fisher’s exact test; Bonferroni adjusted:	*p *=* *0.226	***p *<* *0.0001**	***p *<* *0.0001**

Bold indicates *p* < 0.05. Std resid, standardized residuals of PNA group from χ^2^ test.

### Spontaneous firing rate

**Figure 2.**
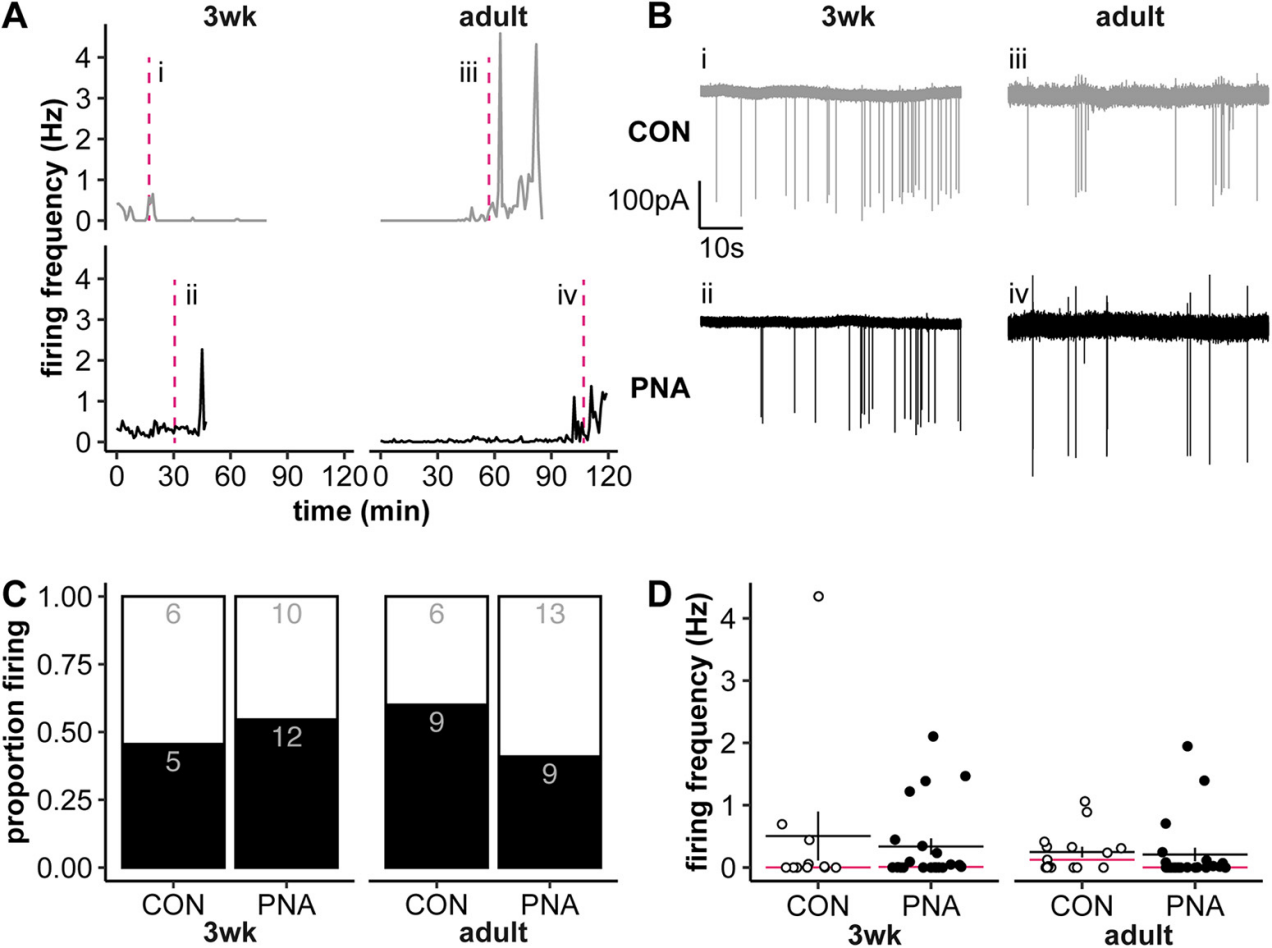
Effect of age and PNA treatment on KNDy neuron firing activity. ***A***, Representative long-term firing patterns (1-min bins). CON are shown on the top in gray, PNA on the bottom in black for each age. The time of the traces shown in ***B*** is designated by the magenta dashed lines in panel ***A***. ***B***, Examples of raw firing data (60 s) from the areas indicated in ***A***, details as in ***A***. The selected 60-s bins are representative of the mean firing rate of each group. ***C***, Proportion of cells with a firing frequency >0.005 Hz (black bars) versus <0.005 Hz (white bars); numbers are cell counts in each group. ***D***, Individual values (CON open symbols, PNA black symbols) and mean ± SEM and median (magenta line) for firing frequency across the duration of long-term recordings. Statistical parameters reported in Table 3.

To determine how age and PNA treatment alter the firing activity of KNDy neurons, we conducted targeted, long-term extracellular recordings of Tac2-GFP-identified neurons in the arcuate nucleus of the hypothalamus. Recordings were obtained from both control and PNA mice at either 3wks of age or in adulthood. These neurons exhibited firing patterns consistent with episodic activity (representative traces in Fig. 2*A*,*B*). About half of recorded Tac2-GFP neurons were quiescent (defined as <0.005 Hz). The proportion of quiescent neurons did not vary with age or treatment (Fig. 2*C*; Breslow-Day test for independence and Mantel-Haenszel χ^2^ test (Simonoff, 2003) statistical parameters reported in Table 3; 3wk-CON *n* = 11 cells from 7 mice in 5 litters, 3wk-PNA *n* = 22 cells from 12 mice in 6 litters, adult-CON *n* = 15 cells from 10 mice in 6 litters, adult-PNA *n* = 22 cells from 13 mice in 8 litters). Neither age nor PNA treatment affected the mean firing frequency of Tac2-GFP neurons over the recording period (Fig. 2*D*).

**Table 3:** Statistical parameters for firing activity (**Fig. 2**)

Parameter	Breslow–Day test for independence	Mantel–Haenszel χ^2^	
Proportion firing	χ^2^ = 1.285, df* *=* *1, *p *=* *0.257	χ^2^ = 0.069, df* *=* *1, *p *=* *0.7932	
Parameter	Age	PNA treatment	Interaction
Firing frequency	Diff −0.193 [CI −0.537, 0.151]	Diff −0.104 [CI −0.448, 0.240]	Diff 0.130 [CI −0.558, 0.819]
	*F*_(1,66)_ = 1.251; *p *=* *0.267	*F*_(1,66)_ = 0.362; *p *=* *0.549	*F*_(1,66)_ = 0.143; *p *=* *0.707

Independence of treatment and age with firing proportion was assessed with Breslow–Day test. This was followed by the Mantel–Haenszel χ^2^ test with continuity correction to determine the effect of treatment on firing proportion when controlling for age (Simonoff, 2003). A two-way ANOVA was conducted for firing frequency.

Figure 2 includes cells that were quiescent; quiescence is likely an important part of the biology of these cells given their postulated role as a component of the GnRH pulse generator. It is possible that either PNA treatment or development modify the degree of firing during active periods. When the firing frequency of just the spontaneously active (≥0.005 Hz) cells is compared, however, there were still no differences (two-way interaction: *F*_(1,29)_ = 0.351, *p *=* *0.558; effect of treatment: *F*_(1,29)_ = 0.183, *p *=* *0.672; effect of age: *F*_(1,29)_ = 2.460, *p *=* *0.128). Of note, the variation in firing rate for cells recorded from the same mouse is similar to that observed when all data are considered. Specifically, for the 24 mice from which two or three cells were recorded, the mean difference in firing rate between the maximum and minimum firing cells was 0.53 Hz (±0.20 Hz; median: 0.19 Hz), and the mean coefficient of variance for the firing rate of cells from the same mouse was 94.8% (±15.9%; median: 71.0%). Thus, using multiple cells from an animal does not artificially lower variance, nor is the variation in firing rate observed fully attributed to individual variation.

### Response to senktide

**Figure 3.**
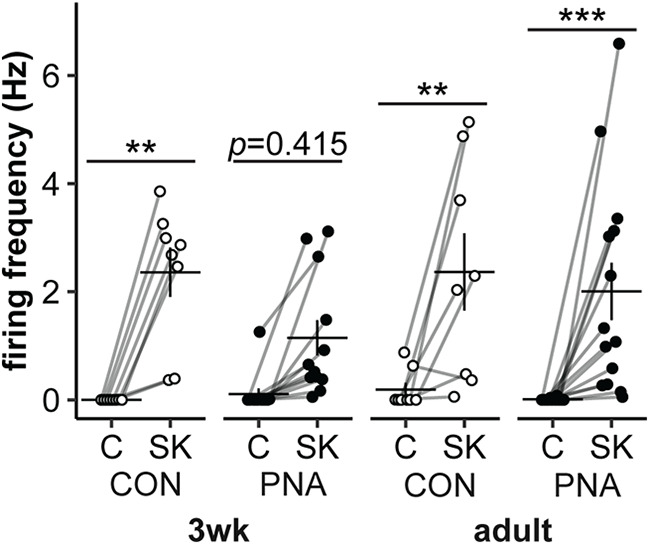
Senktide activates KNDy neuron firing activity. Individual values and mean ± SEM of firing rate during 5-min control (C) and senktide (SK) periods. CON mice are shown in open symbols, PNA mice in black symbols; **p ≤ 0.01, ***p ≤ 0.001. Statistical parameters in Table 4.

To verify viability of quiescent cells, the neurokinin-3 receptor agonist senktide was added at the conclusion of a subset of spontaneous recordings (3wk-CON *n* = 8 cells from 6 mice in 5 litters, 3wk-PNA *n* = 12 cells from 8 mice in 4 litters, adult-CON *n* = 8 cells from 6 mice in 3 litters, adult-PNA *n* = 14 cells from 10 mice in 6 litters). Only cells with spontaneous activity or action currents following senktide addition were included in the dataset. While performed as a quality check, this test is also biologically relevant as senktide activates firing activity of Tac2-GFP neurons (Ruka et al., 2013), and it is possible that age and treatment alter this. Tac2-GFP neurons responded to senktide with an increase in firing frequency (Fig. 3; statistical parameters in Table 4, main effect of time, *p* < 0.0001). This increase was evident in adult-CON (*p *=* *0.007), adult-PNA (*p *=* *0.001), and 3wk-CON (*p *=* *0.003), yet there was not a significant increase for the 3wk-PNA (*p *=* *0.415). This suggests that PNA treatment may alter the development of the response to senktide in KNDy neurons.

**Table 4:** Statistical parameters from three-way mixed model ANOVA assessing the effect of age and PNA treatment on the KNDy cell response to senktide (**Fig. 3)**

Effect	Statistic	Generalized η^2^
Senktide	*F*_(1,38)_ = 52.35, *p *<* *0.0001	0.395
Age	*F*_(1,38)_ = 0.7515, *p *=* *0.391	0.010
PNA treatment	*F*_(1,38)_ = 2.235, *p *=* *0.143	0.030
Senktide × age	*F*_(1,38)_ = 0.5459, *p *=* *0.465	0.007
Senktide × PNA treatment	*F*_(1,38)_ = 2.051, *p *=* *0.160	0.025
Age × treatment	*F*_(1,38)_ = 0.2633, *p *=* *0.611	0.004
Senktide × age × PNA treatment	*F*_(1,38)_ = 1.179, *p *=* *0.284	0.015
Bonferroni’s multiple comparisons test	Mean diff (Hz), 95% CI of diff	Statistic
SK 3wk-CON – C 3wk-CON	Diff 2.36, [CI 0.59, 4.13]	*t*_(38)_ = 4.055, ***p *=* *0.003**
SK 3wk-PNA – C 3wk-PNA	Diff 1.04, [CI −0.41, 2.49]	*t*_(38)_ = 2.192, *p *=* *0.415
SK adult-CON – C adult-CON	Diff 2.178, [CI 0.40, 3.95]	*t*_(38)_ = 3.743, ***p *=* *0.007**
SK adult-PNA – C adult-PNA	Diff 2.00, [CI 0.66, 3.34]	*t*_(38)_ = 4.540, ***p *=* *0.001**
C 3wk-PNA – C 3wk-CON	Diff 0.11, [CI −1.51, 1.72]	*t*_(76)_ = 0.194, *p *>* *0.999
C adult-PNA – C adult-CON	Diff −0.18, [CI −1.75, 1.39]	*t*_(76)_ = 0.340, *p *>* *0.999
SK 3wk-PNA – SK 3wk-CON	Diff −1.21, [CI −2.82, 0.40]	*t*_(76)_ = 2.223, *p *=* *0.350
SK adult-PNA – SK adult-CON	Diff −0.36, [CI −1.93, 1.20]	*t*_(76)_ = 0.682, *p *>* *0.999
C adult-CON – C 3wk-CON	Diff 0.19, [CI −1.58, 1.95]	*t*_(76)_ = 0.315, *p *>* *0.999
C adult-PNA – C 3wk-PNA	Diff −0.10, [CI −1.49, 1.29]	*t*_(76)_ = 0.207, *p *>* *0.999
SK adult-CON – SK 3wk-CON	Diff 0.01, [CI −1.76, 1.77]	*t*_(76)_ = 0.011, *p *>* *0.999
SK adult-PNA – SK 3wk-PNA	Diff 0.86, [CI −0.53, 2.25]	*t*_(76)_ = 1.825, *p *=* *0.863

The effect size, generalized η^2^, is reported for each effect. Bonferroni multiple comparisons for cells that differ by only one factor. Bold indicates *p *<* *0.05.

### Short-term firing pattern

**Figure 4.**
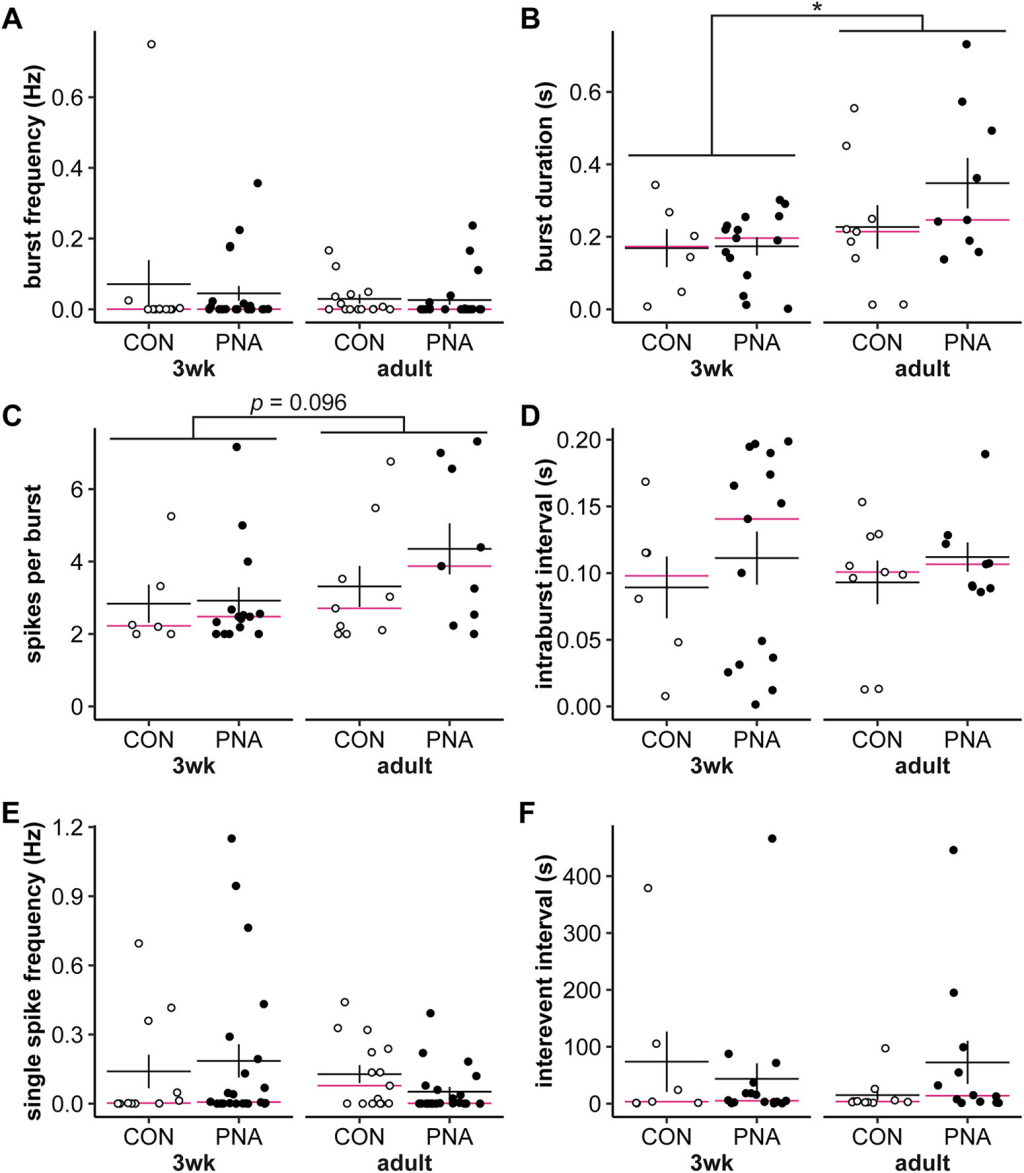
Effect of age and PNA treatment on burst parameters. ***A–F***, Individual values (CON open symbols, PNA black symbols) and mean ± SEM and median (magenta lines) for burst frequency (***A***), burst duration (***B***), spikes per burst (***C***), intraburst interval (***D***), single spike frequency (***E***), and interevent interval (***F***). ***B–D***, Burst parameters are only calculated for cells with at least one burst; 3wk-CON *n* = 6 cells from 5 mice in 4 litters, 3wk-PNA *n* = 15 cells from 10 mice in 5 litters, adult-CON *n* = 9 cells from 8 mice in 5 litters, adult-PNA *n* = 9 cells from 8 mice in 5 litters. ***F***, Calculating interevent interval also requires multiple events, 3wk-CON *n* = 7 cells from 6 mice in 5 litters, 3wk-PNA *n* = 17 cells from 10 mice in 5 litters, adult-CON *n* = 10 cells from 10 mice in 6 litters, adult-PNA *n* = 12 cells from 9 mice in 5 litters; *p < 0.05. Statistical parameters in Table 5.

Examining the average firing frequency over the duration of the recording could obscure changes in the short-term organization of action potentials that may be more relevant for neurosecretion (Cazalis et al., 1985; Dutton and Dyball, 1979). We thus investigated the effect of age and PNA treatment on short-term firing patterns called bursts. (Fig. 4; statistical parameters in Table 5). Because not all cells exhibit burst firing, the n for cells changes for parts B, C, and D, and for part F as detailed in the legend. There were no differences because of age or treatment on any parameter other than burst duration. Burst duration was greater in cells from adults than those from 3wk-old mice (Fig. 4*B*, *p *=* *0.031). An increase in burst duration could occur as a result of more spikes per burst, and/or a longer intraburst interval. Although it did not reach the level set for statistical significance, the increase in burst duration in adults appears to be driven primarily by increased spikes per burst (Fig. 4*C*, *p *=* *0.096) rather than a change in the intraburst interval (Fig. 4*D*, *p *=* *0.911).

**Table 5:** Two-way ANOVA statistical parameters for burst parameters

Property	Age	PNA treatment	Interaction
Burst frequency	Diff −0.030 [CI −0.086, 0.025]	Diff −0.014 [CI −0.070, 0.041]	Diff 0.023 [CI −0.088, 0.134]
	*F*_(1,66)_ = 1.184; *p *=* *0.281	*F*_(1,66)_ = 0.267; *p *=* *0.608	*F*_(1,66)_ = 0.167; *p *=* *0.684
Burst duration	Diff 0.116 [CI 0.011, 0.221]	Diff 0.063 [CI −0.042, 0.168]	Diff 0.116 [CI −0.94, 0.326]
	*F*_(1,35)_ = 5.068; ***p *=* *0.031**	*F*_(1,35)_ = 1.479; *p *=* *0.232	*F*_(1,35)_ = 1.263; *p *=* *0.269
Spikes per burst	Diff 0.954 [CI −0.178, 2.087]	Diff 0.560 [CI −0.572, 1.693]	Diff 0.955 [CI −1.310, 3.220]
	*F*_(1,35)_ = 2.927; *p *=* *0.096	*F*_(1,35)_ = 1.009; *p *=* *0.322	*F*_(1,35)_ = 0.733; *p *=* *0.398
Intraburst interval	Diff 0.002 [CI −0.039, 0.044]	Diff 0.021 [CI −0.021, 0.062]	Diff −0.003 [CI −0.085, 0.079]
	*F*_(1,35)_ = 0.013; *p *=* *0.911	*F*_(1,35)_ = 1.021; *p *=* *0.319	*F*_(1,35)_ = 0.005; *p *=* *0.942
Single spike frequency	Diff −0.073 [CI −0.187, 0.041]	Diff −0.015 [CI −0.129, 0.099]	Diff −0.122 [CI −0.350, 0.106]
	*F*_(1,66)_ = 1.63; *p *=* *0.206	*F*_(1,66)_ = 0.072; *p *=* *0.790	*F*_(1,66)_ = 1.147; *p *=* *0.288
Interburst interval	Diff −14.87 [CI −84.09, 54.36]	Diff 13.72 [CI −55.51, 82.94]	Diff 87.73 [CI −50.71, 226.2]
	*F*_(1,42)_ = 0.188; *p *=* *0.667	*F*_(1,42)_ = 0.160; *p *=* *0.691	*F*_(1,42)_ = 1.635; *p *=* *0.208

Bold indicates *p *<* *0.05.

### Development but not PNA affects expression of key transcripts

**Figure 5.**
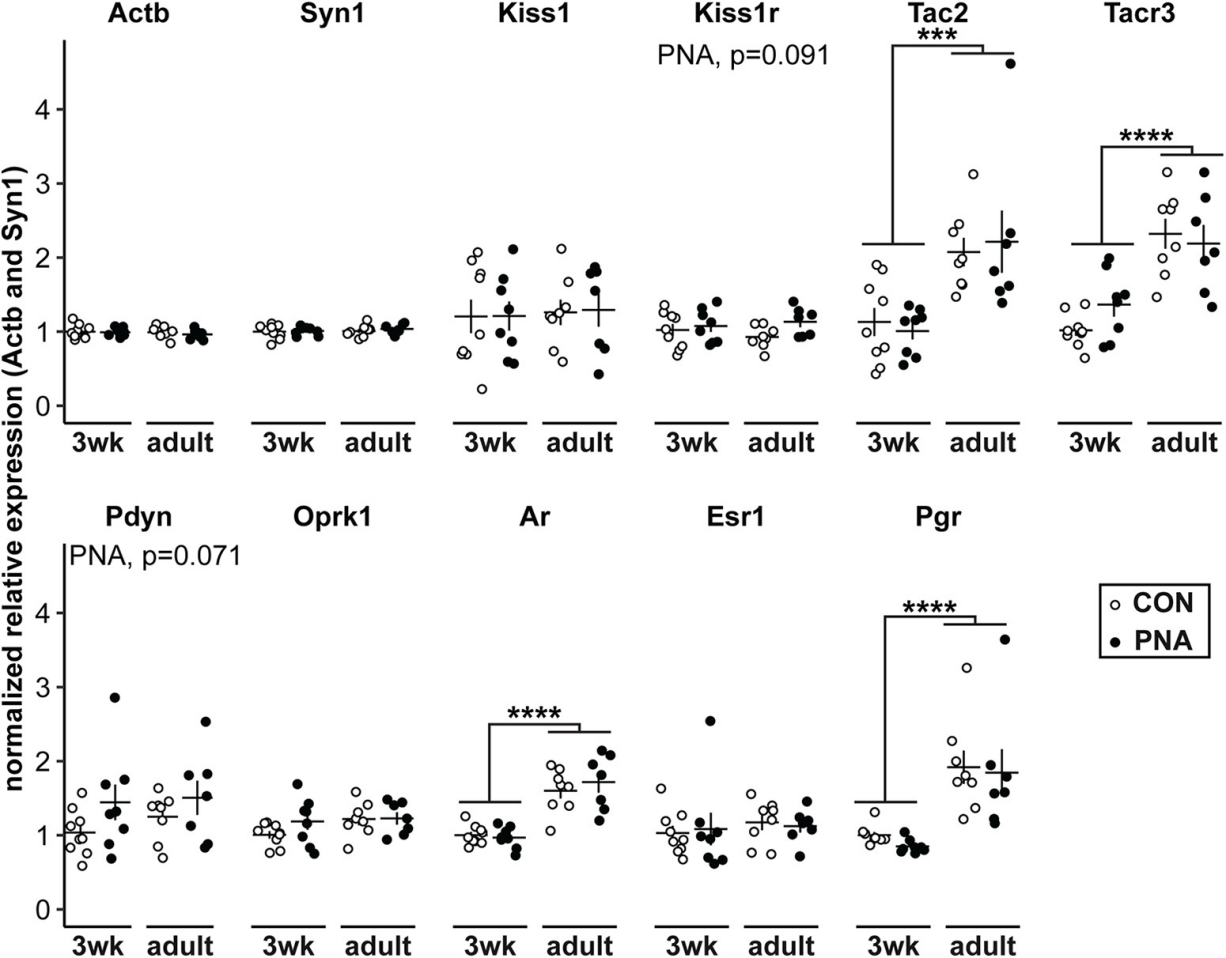
Effect of age and PNA treatment on arcuate nucleus mRNA transcripts. Individual values (CON open symbols, PNA black symbols) and mean ± SEM for *Actb*, *Syn1*, *Kiss1*, *Kiss1r*, *Tac2*, *Tacr3*, *Pdyn*, *Oprk1*, *Ar*, *Esr1*, and *Pgr* mRNA isolated from arcuate nucleus tissue punches. Statistical parameters are reported in Table 6; ***p ≤ 0.001, ****p ≤ 0.0001.

To examine the effects of age and PNA on steroid receptors and KNDy neuron peptides and receptors in the arcuate nucleus, we quantified mRNA expression of androgen (*Ar*), estrogen (*Esr1*), and progesterone (*Pgr*) receptors and of kisspeptin (*Kiss1*), neurokinin B (*Tac2*), and dynorphin (*Pdyn*), and their corresponding receptors (*Kiss1r*, *Tacr3*, and *Oprk1*, respectively). *Tac2* (*p* = 0.0001) and *Tacr3* (*p* < 0.0001) expression were both increased in adults compared with 3wk-old mice (Fig. 5, statistical parameters in Table 6; 3wk-CON *n* = 9 mice, 3wk-PNA *n* = 8 mice, adult-CON *n* = 8 mice, adult-PNA *n* = 7 mice). Similarly, *Ar* (*p* < 0.0001) and *Pgr* (*p* < 0.0001) were increased in the adult arcuate nucleus (Fig. 5). PNA treatment did not alter expression of any transcripts, although there were weak trends for PNA to increase expression of *Kiss1r* (*p* = 0.091) and *Pdyn* (*p* = 0.071; Fig. 5).

**Table 6:** Two-way ANOVA statistical parameters for arcuate mRNA expression

Transcript	Age	PNA treatment	Interaction
*Actb*	Diff −0.018 [CI −0.074, 0.038]	Diff −0.019B2 [CI −0.074, 0.037]	Diff −0.019 [CI −0.131, 0.092]
	*F*_(1,28)_ = 0.436; *p *=* *0.515	*F*_(1,28)_ = 0.466; *p *=* *0.501	*F*_(1,28)_ = 0.126; *p *=* *0.725
*Syn1*	Diff 0.018 [CI −0.038, 0.074]	Diff 0.019 [CI −0.037, 0.074]	Diff 0.019 [CI −0.092, 0.131]
	*F*_(1,28)_ = 0.436; *p *=* *0.515	*F*_(1,28)_ = 0.466; *p *=* *0.501	*F*_(1,28)_ = 0.126; *p *=* *0.725
*Kiss1*	Diff 0.068 [CI −0.358, 0.495]	Diff 0.019 [CI −0.407, 0.445]	Diff 0.028 [CI −0.824, 0.880]
	*F*_(1,28)_ = 0.108; *p *=* *0.744	*F*_(1,28)_ = 0.009; *p *=* *0.926	*F*_(1,28)_ = 0.004; *p *=* *0.947
*Kiss1r*	Diff −0.019 [CI −0.170, 0.132]	Diff 0.129 [CI −0.022, 0.280]	Diff 0.150 [CI −0.152, 0.453]
	*F*_(1,28)_ = 0.068; *p *=* *0.796	*F*_(1,28)_ = 3.062; *p *=* *0.091	*F*_(1,28)_ = 1.038; *p *=* *0.317
*Tac2*	Diff 1.075 [CI 0.584, 1.566]	Diff 0.009 [CI −0.482, 0.500]	Diff 0.264 [CI −0.718, 1.245]
	*F*_(1,28)_ = 20.113; ***p *=* *0.0001**	*F*_(1,28)_ = 0.001; *p *=* *0.97	*F*_(1,28)_ = 0.302; *p *=* *0.587
*Tacr3*	Diff 1.064 [CI 0.707, 1.420]	Diff 0.108 [CI −0.249, 0.464]	Diff −0.476 [CI −1.189, 0.238]
	*F*_(1,28)_ = 37.321; ***p *<* *0.0001**	*F*_(1,28)_ = 0.381; *p *=* *0.542	*F*_(1,28)_ = 1.865; *p *=* *0.183
*Pdyn*	Diff 0.138 [CI −0.224, 0.499]	Diff 0.331 [CI −0.030, 0.693]	Diff −0.153 [CI −0.876, 0.571]
	*F*_(1,28)_ = 0.608; *p *=* *0.442	*F*_(1,28)_ = 3.520; *p *=* *0.071	*F*_(1,28)_ = 0.187; *p *=* *0.669
*Opkr1*	Diff 0.127 [CI −0.044, 0.298]	Diff 0.095 [CI −0.076, 0.266]	Diff −0.170 [CI −0.512, 0.172]
	*F*_(1,28)_ = 2.313; *p *=* *0.139	*F*_(1,28)_ = 1.294; *p *=* *0.265	*F*_(1,28)_ = 1.033; *p *=* *0.318
*Ar*	Diff 0.673 [CI 0.493, 0.854]	Diff 0.041 [CI −0.140, 0.221]	Diff 0.150 [CI −0.212, 0.511]
	*F*_(1,28)_ = 58.323; ***p *<* *0.0001**	*F*_(1,28)_ = 0.215; *p *=* *0.647	*F*_(1,28)_ = 0.720; *p *=* *0.403
*Esr1*	Diff 0.091 [CI −0.195, 0.377]	Diff 0.002 [CI −0.284, 0.288]	Diff −0.105 [CI −677, 0.467]
	*F*_(1,28)_ = 0.428; *p *=* *0.519	*F*_(1,28)_ = 0.0002; *p *=* *0.989	*F*_(1,28)_ = 0.141; *p *=* *0.71
*Pgr*	Diff 0.955 [CI 0.585, 1.325]	Diff −0.114 [CI −0.484, 0.257]	Diff 0.076 [CI −0.664, 0.817]
	*F*_(1,28)_ = 27.92; ***p *<* *0.0001**	*F*_(1,28)_ = 0.395; *p *=* *0.535	*F*_(1,28)_ = 0.045; *p *=* *0.834

Bold indicates *p *<* *0.05.

## Discussion

Changes in the frequency of GnRH release throughout female reproductive cycles are important for fertility. Patients with PCOS often fail to exhibit these changes, instead displaying a persistently elevated LH, and presumably GnRH, pulse frequency. Here, we tested the hypothesis that prenatal exposure to elevated androgens, a model that recapitulates aspects of PCOS, disrupts the hypothalamo-pituitary-gonadal axis in part by changing the firing activity of KNDy neurons in the arcuate nucleus. Contrary to our hypothesis, neither overall spontaneous activity of KNDy neurons nor most burst characteristics were altered by PNA treatment either before puberty or in adulthood. Expression of *Tac2*, *Tacr3*, *Ar*, and *Pgr* mRNA was greater in the arcuate of adult mice as compared with 3wk-old mice, but this expression was not impacted by PNA treatment. These findings suggest that changes in KNDy neuron activity alone are not responsible for the altered LH pulse frequency observed with PNA treatment.

KNDy neurons have been postulated to be the pulse generator for GnRH release (Clarkson et al., 2017; McQuillan et al., 2019). Kisspeptin increases GnRH release (Messager et al., 2005; Glanowska et al., 2014) *in vivo* and in brain slices, and increases GnRH neuron activity (Han et al., 2005; Pielecka-Fortuna et al., 2008) in brain slices. As the putative pulse generator, KNDy neuron activity would be expected to change in manners that reflect the output of GnRH neurons and LH release. GnRH neuron firing rate changes with development and PNA treatment alters the typical developmental trajectory. Specifically, in GnRH neurons from adults, firing rate is elevated in PNA mice (Roland and Moenter, 2011), whereas firing frequency in cells from PNA mice before puberty at 3wks of age is reduced, because PNA treatment blunts the typical peak in firing that occurs near this age in control mice (Dulka and Moenter, 2017). We expected similar effects in KNDy neurons. Consistent with prior studies in adults, many of the KNDy cells that we recorded were quiescent (de Croft et al., 2012; Frazão et al., 2013; Ruka et al., 2013). Surprisingly, neither age nor PNA treatment altered the mean firing frequency of KNDy neurons. Similarly, effects on short-term burst firing were minimal. These observations suggest that both development and prenatal exposure to androgens alter GnRH neuron activity and release via mechanisms other than changing the activity of KNDy neurons.

The elevated LH pulse frequency in patients with PCOS is attributable at least in part to reduced negative feedback actions of progesterone (Pastor et al., 1998). When patients are treated with the anti-androgen flutamide, the suppressive effects of progesterone on LH release are partially restored (Eagleson et al., 2000), suggesting that hyperandrogenism plays a role in this impaired negative feedback. The opposing effects of androgens and progestins is supported by findings in murine brain slices that androgens interfere with progesterone negative feedback on GnRH neuron firing rate (Pielecka et al., 2006) and GABA transmission to these cells (Sullivan and Moenter, 2005). The elevated LH pulse frequency in PNA mice (Moore et al., 2015) may have a similar origin to that in patients with PCOS. Following ovariectomy, LH levels rise in control mice and to a lesser extent in PNA mice (Moore et al., 2015). Administration of progesterone reduces the LH levels in ovariectomized control but not PNA mice, indicative of impaired negative feedback in the latter (Moore et al., 2015). Progesterone may act in part through receptors in the arcuate nucleus to reduce LH pulse frequency, as administration of progesterone receptor antagonists in this brain region reduces the interval between LH pulses following intraperitoneal injection of progesterone (He et al., 2017). Progesterone also reduces the frequency of peaks in KNDy neuron activity measured by GCaMP fluorescence that are correlated with LH release (McQuillan et al., 2019), but whether or not this is a direct effect on KNDy neurons is not known.

A possible alternative mediator of central changes in PNA mice is GABAergic neurons. A subset of KNDy neurons may be GABAergic, although estimates vary on the percentage; up to 50% of KNDy neurons express GAD67 (Cravo et al., 2011), but only ∼10–15% of KNDy neurons co-express the vesicular GABA transporter VGaT (Marshall et al., 2017). PNA treatment increases the frequency of GABAergic postsynaptic currents recorded in GnRH neurons from both adult and prepubertal 3wk-old mice (Sullivan and Moenter, 2004; Berg et al., 2018), which given the excitatory effects of GABA in GnRH neurons can contribute to increased activity. At least some of this increased transmission appears to arise from the arcuate nucleus as appositions between GABAergic neurons in the region and GnRH neurons increase in PNA mice (Moore et al., 2015). Consistent with an involvement in the steroid feedback effects discussed above, PNA treatment reduces the expression of progesterone receptors in GABAergic neurons in the arcuate nucleus (Moore et al., 2015). High-frequency optogenetic stimulation of GABAergic neurons in the arcuate nucleus can stimulate LH release in control mice (Silva et al., 2019). Longer-term chemogenetic activation of these GABAergic neurons also increases LH release, disrupts estrous cycles and decreases the number of corpora lutea (Silva et al., 2019). Together, these results point to GABAergic neurons in the arcuate as a potential steroid-sensitive mediator of PNA treatment on GnRH neuron activity.

It is important to point out that our findings do not rule out a possible role of KNDy neurons in modulating the effects of PNA or age on GnRH and LH release. KNDy neurons are part of an intricate network in the arcuate nucleus, and they project to GnRH distal projections in the median eminence (Yip et al., 2015). In rats, PNA increases the number of arcuate cells immunopositive for kisspeptin and neurokinin B (Osuka et al., 2017) and the relative levels of kisspeptin and neurokinin B mRNA (Yan et al., 2014). In sheep, prenatal testosterone treatment reduced the number of putatively inhibitory dynorphin-immunopositive cells without changing the number of kisspeptin-immunopositive cells in the arcuate nucleus (Cheng et al., 2010). In contrast, following 7 d of estradiol treatment, PNA mice did not differ in the relative mRNA expression of KNDy neuron peptides or receptors in the arcuate nucleus (Caldwell et al., 2015). Similarly, we only observed developmental changes in KNDy neuron peptide and receptor mRNA expression in the arcuate nucleus, but no changes because of PNA treatment. The variation between studies may be attributable to animal models or the examination of mRNA versus peptide.

Prenatal testosterone exposure also alters synaptic connections of KNDy neurons with one another and projections to GnRH neurons (Cernea et al., 2015). It remains possible that even without a change in firing frequency, PNA may alter the amount of kisspeptin and/or other neuromodulators released at a given level of activity, potentially leading to increased GnRH release. The reciprocal connections of the network of KNDy neurons are also thought to be important for their involvement in GnRH pulse regulation, and PNA could alter these dynamics. Neurokinin B increases and dynorphin decreases the activity of KNDy neurons (de Croft et al., 2012, 2013; Ruka et al., 2013), whereas kisspeptin does not affect the firing frequency of other KNDy neurons (de Croft et al., 2013). The effects of neurokinin B and dynorphin signaling are modulated by steroidal milieu (Ruka et al., 2016). Although we did not observe effects of PNA on mRNA expression within the whole arcuate nucleus, our study does not address the possibility that gene expression, including that of receptors for KNDy peptides, may be altered specifically within KNDy neurons. Intriguingly, in the present study, senktide, a neurokinin-3 receptor agonist, was less effective at eliciting an increase in firing frequency of KNDy neurons from 3wk-old PNA mice. This suggests that PNA disrupts the development of this network.

A key feature of patients with PCOS is a persistently elevated LH pulse frequency that is most similar to the mid-to-late follicular phase (McCartney et al., 2002). Although we often recorded cells for at least 60 min, these recordings were not of sufficient length to characterize rigorously the frequency and duration of peaks and nadirs in firing activity. It is plausible that the firing activity of KNDy neurons during a peak in activity does not differ with age or PNA treatment, but that the frequency of these peaks may be increased in adult-PNA mice, leading to the elevated GnRH and LH pulse frequency. In this regard, the frequency of peaks, but not mean firing rate, was altered in KNDy neurons from adult males by orchidectomy and steroid replacement (Vanacker et al., 2017). Because PNA mice fail to exhibit typical estrous cycles and remain persistently in a diestrus-like state based on vaginal cytology, we specifically compared their firing frequency to that of cells from diestrous control mice. The frequency of peaks in calcium activity of KNDy neurons across the estrous cycle was not different from metestrus to diestrus to proestrus (McQuillan et al., 2019). In contrast, KNDy cells from mice in estrus exhibited a markedly decreased frequency of these peaks, postulated to be due in part to the negative feedback effects of progesterone (McQuillan et al., 2019). It is thus possible that a difference in firing rate of KNDy neurons would be observed on estrus that could be attributable to impaired progesterone negative feedback in adult-PNA mice. Our choice to record on diestrus was based not only on the practical consideration that PNA mice are often in persistent diestrus, but also on the observed increase in LH pulse frequency in PNA mice during this stage (Moore et al., 2015). The lack of difference in KNDy neuron firing rate in the present study thus supports the postulate that this increased episodic LH release arises from other cells, or is disrupted by the brain slice preparation.

The work presented here indicates that the elevated GnRH neuron firing frequency and LH pulse frequency associated with PNA cannot be solely explained by changes in arcuate KNDy neuron firing frequency or bursting patterns. PNA may also alter the development of the stimulatory effects of neurokinin-3 receptor activation on KNDy neuron activity, disrupting network dynamics. Our work points to the importance of the broader network of neurons within the hypothalamus, including GABAergic cells, as mediators of the effects of hyperandrogenism on the output of the hypothalamic-pituitary-gonadal axis.

## Synthesis

Reviewing Editor: Tod E Kippin, University of California Decisions are customarily a result of the Reviewing Editor and the peer reviewers coming together and discussing their recommendations until a consensus is reached. When revisions are invited, a fact-based synthesis statement explaining their decision and outlining what is needed to prepare a revision will be listed below. The following reviewer(s) agreed to reveal their identity: Jonathan VanRyzin.

The present study investigates the impact of perinatal androgen (PNA) treatment on the neural substrates controlling female reproductive cycle in adulthood by testing the hypothesis that PNA-induced changes in gonadotropin-releasing hormone (GnRH) neurons might be mediated via alterations in their upstream regulators, the kisspeptin neurons. The manuscript present electrophysiological and molecular experiments testing this hypothesis with the conclusion that this is not the case. As such, a hypothetical mechanisms for PNA alterations of GnRH disruptions is ruled out. This negative result is viewed as an important addition to the field.

The paper was evaluated by two independent reviewers whom were both highly positive about the potential significance of the manuscript but did raise some concerns that need to be addressed as indicated below.

Reviewer # 1

Despite reporting essentially negative data, I think this is a valuable and rigorously-carried out study that adds to our understanding of what changes in the GnRH system, GnRH cells and their afferents, may (or may not) underlie reproductive deficits in the PNA mouse model of PCOS. Nonetheless, I do have some questions for the authors about the data, as well as caveats, that need to be clarified:

1. Recognizing the convention in electrophysiological studies of considering the number of recordings from individual cells as the sample size, I still wonder whether any of the variation seen in responses (e.g., Fig 2D, PNA groups) could be attributed to individual differences among the animals? From in the diestrous data (Fig. 1D), it looks like some animals are more disrupted than others, and if the authors had performed LH pulse bleeds they also may have varied by pulse frequency. In any case, it would be good for authors to consider whether individual animal variation might have masked the detection of significant differences between control and PNA cells in parameters studied. And if there is variation among animals, then perhaps if more cells were added to the analyses (e.g., Fig. 2D), significant differences in firing frequency might arise between control and PNA groups.

In addition, for Figure 2D, it looks like both responding and non-responding cells were included in the comparison of firing frequency. Was a comparison made between groups using the responding cells alone?

2. A significant caveat to the PCR analysis reported is in the use of whole ARC micropunches (containing more than just KNDy cells) so that any KNDy-specific changes in mRNAs (with the exception of KNDy peptide mRNAs), may be masked by inclusion of non-KNDy cells that express these receptors. This is not a fatal flaw in my view, since the PCR measurements are not the major finding of the paper; it just needs to be acknowledged in the discussion.

3. The methods state that either Senktide or potassium was used to test the viability of cells. Three-week old PNA mice did not show a significant increase in activity after Senktide treatment, which the authors postulate may be due to PNA treatment altering their developmental response to the agonist. Since Senktide treatment was unable to confirm the viability of these cells, was potassium used?

4. Finally, we are a bit confused about Figure 4A - it looks like the frequency for most of the cells with burst firing is at zero, but then they are able to get data from these cells for B and C. And how can the interval in D also be zero for some of these cells when the authors say they only included cells with more than one burst event?

Reviewer # 2

This manuscript tests an interesting hypothesis-that prenatal androgen treatment alters the firing frequencies of KNDy neurons in the arcuate nucleus-in order to identify a potential mechanism by which GnRH neuron activity is disrupted in this experimental model. The question is very well framed; the authors clearly identified their candidate mechanism and they outlined two key predictions that followed. Overall, the authors found that the data did not support their hypothesis and instead propose new avenues for research moving forward.

Given that this manuscript largely includes “negative” data, I really enjoyed the way the manuscript was laid out and written. By starting with the original hypothesis and predictions, I was able to easily follow the logic and come to similar conclusions.

After all, this is most often how science is done-identify a hypothesis, test the predictions, and accept/revise/reject the initial hypothesis.

I have no real major concerns with the manuscript, only a few minor comments.

1. At the beginning of the results, it would be helpful to have a quick re-introduction into the experimental paradigm. Simply a line or two describing the injection timeline in reference to the phenotypic measures being described would suffice. This could (but not necessary) also include a small timeline panel in Figure

2. Were quiescent and non-quiescent cells included in the analyses of spontaneous firing rate? If so, is there any difference when analyzing only the non-quiescent cells?

3. What was the rationale for when the representative traces in Figure 2B were chosen? While the data in Figure 2D show convincingly no difference in firing frequency, the traces in 2B look (by eye) to have differences in frequency between i. and ii. and burst firing between iii. and iv.

4. The black bars indicating mean +/- SEM are hard to see in some cases, particularly with Figure 5 with how tightly plotted the individual datapoints are. The authors might consider choosing an alternative color to indicate mean and SEM throughout, much like they did with the magenta median line.
