# Supplementary material for: Prenatal Androgen Treatment Does Not Alter the Firing Activity of Hypothalamic Arcuate Kisspeptin Neurons in Female Mice
Source: eNeuro. 2021 Sep 29;8(5):ENEURO.0306-21.2021. doi: 10.1523/ENEURO.0306-21.2021 (PMC8482853; doi:10.1523/ENEURO.0306-21.2021)
Supplement: Extended Data 1 — Code used to detect and analyze events in Igor Pro (coding-project) and for data analysis in R (PNA-KNDy). Download Extended Data 1, ZIP file. [file enu-eN-NWR-0306-21-s01.zip › extended-data-1/coding-project.zip!/coding-project-vbwregions/NEW INSTALL INSTRUCTIONS 20191213.docx]

1 Installation

Delete all the old aliases in your Wavemetrics folders (Igor Procedures and User Procedures). Unzip the “td analysis.zip” file. Make an alias to the folder. Move the alias to the Igor Procedures folder in your Documents/Wavemetrics folder. Use the folder in your Documents folder, not the Wavemetrics folder in the applications folder.

If you have compilation errors, it may be necessary to delete the “include” statements from the Procedure Window.

This is how you get to the Procedure Window:
