# Supplementary material for: Prenatal Androgen Treatment Does Not Alter the Firing Activity of Hypothalamic Arcuate Kisspeptin Neurons in Female Mice
Source: eNeuro. 2021 Sep 29;8(5):ENEURO.0306-21.2021. doi: 10.1523/ENEURO.0306-21.2021 (PMC8482853; doi:10.1523/ENEURO.0306-21.2021)
Supplement: Extended Data 1 — Code used to detect and analyze events in Igor Pro (coding-project) and for data analysis in R (PNA-KNDy). Download Extended Data 1, ZIP file. [file enu-eN-NWR-0306-21-s01.zip › extended-data-1/coding-project.zip!/coding-project-vbwregions/documentation/td analysis/FileFormat_2x71/DataFormat_v9.doc]

PatchMaster v2.21, 07-Sep-2007

# Data Format

In this chapter we describe the general structure of the files generated by *PatchMAster*.

## Data Files

*PatchMAster* generates up to 5 files, when a data file is created:

1. The *Data* file (file extension *.dat).
2. The *Stimulus Templates* file (file extension *.pgf).
3. The *Acquisition Parameters* file (file extension *.pul).
4. The *Notebook* file (file extension *.txt).
5. The *Amplifier* file (file extension *.amp).

Except for the raw data file and the Notebook file, all other files have a *Tree* structure. The entire trees are kept in memory, whereas the raw data traces are always loaded from disk, when needed.

### Stimulation Template: Stim

Stores the stimulation protocol. The structure of the *Stimulation File* (extension “pgf”) is defined by the *Definition Module* “Stim.de”. The *Stimulation File* has a tree structure:

| **Record** | **Description** |
| --- | --- |
| Root | Version number |
| Stimulation | Description of an ensemble of pulse patterns; e.g., I-V curve |
| Channel | Combines the definition for one output (DAC) and one input (ADC) trace |
| Segment | Individual segment of a pulse pattern |

Stimulation files can be loaded into the *Pulse Generator*. In fact, the *Pulse Generator* files for the stimulation protocols used during the experiments have the same data structure as the PGF-files, which belong to the recorded data. In this way it is possible to exactly repeat an experiment by using a copy of a PGF-file as *Pulse Generator* file.

### Data Description: Pulsed

Stores parameters, such as gain, capacitance, etc. The pointer to the data stored in the raw data file is also contained in this file. The structure of the *Pulsed File* (extension “pul”) is defined by the *Definition Module* “Pulsed.de”. The *Pulsed File* has a tree structure:

| **Record** | **Description** |
| --- | --- |
| Root | Version number, text, time, file name |
| Group | Larger section of an experiment; e.g., cell or patch |
| Series | Description of an ensemble of sweeps |
| Sweep | Description of a sweep, i.e. one collection of traces |
| Trace | Description of an individual data trace |

A graphical template of the *Pulsed File* (*Tree*) is shown in the *Replay* window. It contains information necessary to reconstruct the experimental conditions as the data were recorded.

### Amplifier file: AmplTree

This file is only generated, when multiple amplifiers are active (EPC9 Double and Triple, etc.). It contains the amplifier state records of each amplifier for every Series. The structure of the *Amplifier File* (extension “amp”) is defined by the *Definition Module* “AmplTree.de”. The *Amplifier File* has a tree structure:

| **Record** | **Description** |
| --- | --- |
| Root | Version number, description of the tree size |
| Series | Collection of all amplifier state records of one Series |
| Amplifier State | The amplifier state record |

### Raw Data

This raw data file is a continuous data stream. Each data point is a 16-bit signed integer or 32-bit IEEE real as defined in the respective PGF-template. When a sweep is stored, *PatchMAster* stores the various traces (if available) sequentially as defined in the respective PGF-template. Traces with leak pulses are stored leak-subtracted. The leak traces are normally stored after the traces themselves.

Very long traces with “continuous” segments may be stored as interleaved blocks. The size of such a block is given in the “InterleaveSize” field (in bytes) of the Trace record, the distance to the next block in the “InterleaveSkip” field (in bytes).

### Notebook File

The notebook file is a standard ASCII text file with line breaks.

## The Tree Format

The idea of *PatchMaster* is to order the data of an experiment in *Trees*. The trunk of the tree (*Root*) is the main descriptor of a data file (it could, for example, correspond to one cell or the entire experiments of one day). The next level is the *Group*. This level can be defined by the user to identify data that belong together. An example would be to open a new group for each patch. The group may contain several families of records. Such a family (e.g., records of a current–voltage relationship) is called *Series*. The individual records of a family are called *Sweeps*. Finally, each sweep may be composed of *Traces* (at the moment, there are two traces per sweep). The latter are not yet part of the data tree (it is planned to allow for more traces in the future). A copy of this data tree is accessible to the user throughout the experiment (so one has an overview of what was recorded, and one can immediately edit the entries (e.g., discard bad records).

The following is a description of the *Tree* format. *Appendix III* has a source code listing for a program that shows how to scan and load a *Tree* file. The code is commented and can easily be translated to other languages.

An example tree with 4 levels can be diagrammed as follows:

There is only one record of level 0, the root record. The above tree has four levels, the root (level 0), levels 1 and 2, and the leaf nodes (level 3).

The format of a tree stored to a file is as follows:

1. Magic number: 054726565 (hex) = “Tree”
2. Number of levels
3. Level sizes, one per level
4. Tree records, top down, left-to-right. Each record has the format:

- Record contents
- Number of children

All of the values (except the record contents) are INT32 values, i.e., 32-bit (4 bytes) values.

**Note 1**	Check the record sizes in the file headers. The record sizes may differ from what you are expecting, e.g., because the file has been created by an older program version which used fewer fields than it is currently using, or a newer version with additional fields**. You must use the record sizes stored in the files themselves.**

**Note 2**	The “Magic” number will be 065657254 (hex) = “eerT”, if the file was written on an operating system with opposite byte ordering (e.g. written under MacOS, read under Windows). In that case, appropriate byte swapping has to be performed (highest -> lowest, etc.), when the data is read in.
