# Supplementary material for: Prenatal Androgen Treatment Does Not Alter the Firing Activity of Hypothalamic Arcuate Kisspeptin Neurons in Female Mice
Source: eNeuro. 2021 Sep 29;8(5):ENEURO.0306-21.2021. doi: 10.1523/ENEURO.0306-21.2021 (PMC8482853; doi:10.1523/ENEURO.0306-21.2021)
Supplement: Extended Data 1 — Code used to detect and analyze events in Igor Pro (coding-project) and for data analysis in R (PNA-KNDy). Download Extended Data 1, ZIP file. [file enu-eN-NWR-0306-21-s01.zip › extended-data-1/coding-project.zip!/coding-project-vbwregions/documentation/td analysis/TD_AnalysisManual 201303117 (mip-305986's conflicted copy 2016-04-29).doc]

Chapter 1: Installing TD Analysis

Please note, filenames and folder names are in quotes to minimize confusion with long names.

Make sure Igor is up to date (current version on 17 March 2013 is Igor 6.31).

Unzip “TD analysis” file. Add the date to the folder name so you can keep track of version. Inside the TD analysis folder are two folders named “link to igor procs” and “link to user procs”. Make ALIASES (or shortcuts in the Windows world) of both of these.

Igor makes a folder called “Wavemetrics” that will be found in your “documents” folder (Mac) or “my documents” (PC). Inside the “Wavemetrics” folder, open the “Igor Pro 6 user files” folder. In there are folders called “Igor Procedures” and “User Procedures”. MOVE the aliases you created to the appropriate folder. Drag or copy the “link to igor procs” alias to the “Igor Procedures” folder; similarly, drag the “link to user procs” alias to the “User Procedures” folder.

Go back to the “TD analysis” folder and open the “link to igor procs” folder. COPY the file “IVtiming.txt”. MOVE the copy to “Igor procedures” folder inside the “Wavemetrics” folder (the one in your “documents” folder). RENAME copied file to remove the ‘copy’ and the space before copy. The name must be “IVtiming.txt”. Capitalization is important.

NOTE the “IGOR pro” folder in the applications folder contains folders with the same names as above BUT these should NOT be modified. Instead make sure all modifications are done in the “Wavemetrics” folder in the “documents”/”my documents” folder.

Occasionally, a release of the analysis routines will include multiple versions of a procedure file. In this case, please complain to Tony at [tony.defazio@gmail.com](mailto:tony.defazio@gmail.com), and he will send you a cleaned-up version. In the meantime, you can survive this by “killing” the procedure files that are generating the errors. When Igor starts up, it compiles all procedures it finds in the “Igor Pro 6 User Files” folder and subfolders. If multiple versions exist, it throws an error dialog box that says “name already exists as a macro” or “name already exists as a function”. Simply click the “edit procedure” button. Close the procedure window. Igor throws a dialog that asks if you want to hide or kill the procedure window. Kill it without remorse. The alternative is to delete the offending file in the “td analysis” subfolders. Check the folders to see if there are more than one version, e.g. “tonys_procs v2.6.ipf” and “tonys_procs v2.7.ipf”. You can delete the versions with the smaller numbers and re-run Igor. This really should not happen anymore, but this situation can arise in older releases.

**Chapter 2 Importing your data from Patchmaster to Igor**

The software you installed in Chapter 1 makes available a series of macros in Igor Pro. To access these, start Igor, click on the Macros menu, and select “makeBlastPanel”.

Once makeBlastPanel has run, a panel appears with a series of tabs. The first tab is Heka. In this tab there is a button labeled ‘Open PM bundled file’. Click on this to open a file dialog box and navigate to a data file (i.e., “filename.dat”).

The TD analysis routines create many new waves within Igor to facilitate analysis. It is thus necessary to keep the base filename as short as possible. This can be done with an 8 digit date code plus a letter to distinguish between cells on that day. For example, “20130311a” would indicate the first cell recorded on March 11, 2013. The TD analysis routines will issue a request to shorten your file name if you have exceeded 9 characters. You can put anything you like for the filename, if it is under 10 characters but a little thought now will help you keep your data and your waves in Igor organized.

HEKA data file structure in brief

PM files contain experiments, groups, series, sweeps, and traces in a tree like structure compressed into a single “filename.dat” file. You should create a new PM bundled file for EACH cell you record. An example tree as viewed in PatchMaster is shown in Figure X. This .dat file contains 3 groups in experiment 1 (far left blue boxes, E1 1, E1 2, and E1 3). At this time, the TD analysis routines do not acknowledge the first branch, the experiment tree (the routines read all data, but assumes everything is in E1, which it is if you start a new PM file for each cell). If you are running a particularly complex experimental design that requires multiple experiment branches of the data file, please email [tony.defazio@gmail.com](mailto:tony.defazio@gmail.com) and I will begin working on a fix. TD analysis recognizes all the remaining branches of the tree (i.e., groups, series, sweeps, and traces). A single .dat file might contain multiple “groups”. In turn, each “group” contains a number of “series”, and each “series” consists of “sweeps”. Each “sweep” contains a “trace” of each channel recorded. A sweep is similar to an “episode” in pClamp and usually consists of sequence of voltage or current measurements obtained at a defined frequency (e.g. 10 kHz) Understanding how TD analysis displays these data can greatly speed up your ability to examine your files.

Skipping to new section so we can make some progress in the voltage clamp analysis window!!!

Figure X.

In the HEKA tab of BlastPanel, the very first column of listboxes (indicated by red arrow in figure 1) helps you navigate the file structure. By default, when your .dat file is loaded, all sweeps from the first group are displayed. If you click on a group (say “g1”), all series (and thus all sweeps and traces) in that group?? are displayed in “Analysis Graph 1”. If you want to view series 2 from group 3, first click on g3 then click on s2, Individual sweeps can be displayed by clicking on one of them. Examples? AG1 for group, then series 2 G3, then s2? If we are going to get this detailed we’ll want to decide on a data set for example first (still worried ocvm will be hard, and we want them doing passive…)

Which trace is display in Analysis Graph 1 is determined by the selection in the traces listbox. By default, trace 1 is shown (there was another default above…). For example, if you have multiple traces (i.e., if multiple amplifier channels were used to record data, such as Im and Vm of cell1 and Im and Vm of cell 2, traces 1-4), and you select trace 1 then click on a series, only trace 1 of each sweep will be displayed. Note that HEKA will allow you to assign any acquired variable to any trace. Thus, trace 1 will not always be “Imon 1” unless you design your .pgf patterns appropriately. THIS IS REALLY RECOMMENDED. In this example, I assigned “Imon 1” to trace 1, “Vmon 1” to trace 2, “Imon 2” to trace 3, and “Vmon2” to trace 4. An example pattern generator file is shown in Figure Y. IT WOULD HEL sorry it would help to show a PGF and associated expt tree and Blast panel. Again we need to make a good choice for this.

Figure Y. Example pulse generator file for recording four traces.

All of the individual traces for all the sweeps and series for a given group are available for selection in the second column of list boxes, labeled “AllPMwaves”. Selecting one of these traces will cause it to be displayed in analysis graph 1. If you click the button “Import” near the bottom of the panel, the selected trace appears in the “imported waves” listbox to the right. Event analysis requires the waves of interest to be listed in the “imported waves” listbox. Is there an easy way to import all waves and if so should it be mentioned here?

Chapter 3: Data analysis: Voltage Clamp

One thing that I can’t remember if I mentioned earlier is the ability to average data before analysis. Chu does a lot of stuff where he is measuring small things and needs to do this. Likewise on the ramp studies we are averaging 3-5 runs to reduce noise, as we did for the series 2.

Need some lead in to what this is!

1. In the HEKA tab, open 26Nov07_2.dat by clicking the “Open PM bundled file” button. All data will import automatically for VC and CC analysis. All data traces will appear in the blue box and all command protocols in the yellow box. This is an example from a specific study-it should be introduced in a way that makes this clear. Maybe different margins? This is indented but that is really all that suggests this is different.

I’m thinking of my physics book, which had clearly identified sample problems

FIGURE 1:

2. Select a series to analyze. Let’s try series 3. Click “s3” in the Series list box (indicated by the red arrow above). Now you should see the data and voltage protocol only from this series in analysis graph one?. The analysis will operate on whatever data is selected on this page displayed in analysis graph 1?. It is possible to select a group, series, sweep or trace for analysis.

3. Let’s make an IV curve. Click on the VC tab (green arrow in figure xx above). Now you should see the VC analysis tools (Figure yy below).

4. Click on the “Cursors” button to display the vertical lines in “Analysis graph 1”. The red cursors are for baseline adjustment, the blue for series resistance estimation (if you have a test pulse in your protocol, which we highly recommend but isn’t in this example-should we change this to an example with SR adjustment?), and the green lines are for the data analysis range. Slide the baseline and series resistance (red and blue) lines to the far left using the sliders in the VC tab (indicated by red arrow on Figure zz –note you won’t see this arrow on your screen). Use the sliders to move the green cursors to the region of interest. Your screen should look like this Figure aa

4alt. Alternatively, you can load cursor settings from the list box. Scroll down to “minimum” DOES NOT EXIST haven’t had time to start up just reading for clarity(it should right below final2, let me know if it is still missing STILL MISSING BUT WAS ABLE TO ADD using save button), and select it. Note that once you have settings you really like, you can save them by clicking the “save” button for future use.

Now click the “measure” button. There are four variants: “mean” measures mean between the cursors, “min” takes the minimum, “max” takes the maximum, and “smart” uses a simple algorithm that guesses what you are looking for (it takes the larger of the minimum and the maximum for each sweep, useful for IVs that cross zero and you can’t use the mean).

Smoothing invokes a boxcar average of the data between the green? cursors before the measurement. Keep track of what you do so you can be consistent as smoothing here alters neither the raw nor the imported data; the smoothing occurs in a copy of the relevant data, which is subsequently deleted. Series R applies a series resistance correction to the voltage based on the absolute current and Ohm’s law. The Series R value is either calculated from the trace itself (if enabled with the blue cursors) or using a value that you enter in MΩ (enter 10 for 10 MΩ). Enter 0 if you don’t want any correction. Will it be possible to select multiple data analysis parameters (mean, max)? Or will you just re run if you want multiple parameters reported? For max/min, is it possible to make it average for a window of xx points around a peak so you don’t just get a noise spike?

5. Click measure or plot? and the following graph will appear.

6. Currently the data are stored in “IVcorrected” and “IVxcorrected”. If you want to save these waves, you need to rename them. The next version will do this for you.
7. The software will report (in the history window) the reversal potential if the trace crosses zero. What about cases in which Vrev is where two lines cross (eg. Before and after drug x that modifies current) like we did in GABA paper?

What about the plot button?Shows a different graph that needs a bit o explaining. Got some splainin to do?

Also if imported waves box isn’t going to be used maybe kill it and clear imported waves button?

For min and max, is this a single point or is an average of a range? I’d prefer the latter for something like an IA measurement. One point is just so one pointy. OK see current comment above, one thing is consistent!

RAMPS

1. Now click on series 2 in the HEKA tab. The raw data appear in the top graph and the voltage ramp created from the information in the protocol is shown in the bottom graph. Click on the VC tab to analyze the data that appear in these graphs

2. There is some weirdness in the HEKA protocols, which I am resolving with their programmers (see the extra stuff at the end of the voltage protocol). Also, if the protocols change (i.e. added number of sequences), my routines will fail to create a waveform from the protocol. Please email if you encounter this bug: [r.defazio@miami.edu](mailto:r.defazio@miami.edu) Needs updating
3. Once in the VC panel, click on the cursors button and move the red and blue cursors to the far left (or select the “minimum” cursor setting from above). Move the green cursors to the beginning and end of the ramp as below. (FYI the minimum I created worked).

4. Click on the “plot” button to create a plot of the membrane current as a function of the voltage ramp. All the sweeps in the blue graph (analysis graph1) are plotted in the new graph as a function of the voltage in Analysis Graph 2. Sweet. Measure doesn’t seem to work with this-should it- everything is -65.57 V Right now, the Measure button only operates on Analysis Graph 1 and it plots vs. data in Analysis Graph 2. I’m working on setting it up to do a time course analysis. These each have unique names so you don’t have to rename them as in the IV data.
