# Supplementary material for: Prenatal Androgen Treatment Does Not Alter the Firing Activity of Hypothalamic Arcuate Kisspeptin Neurons in Female Mice
Source: eNeuro. 2021 Sep 29;8(5):ENEURO.0306-21.2021. doi: 10.1523/ENEURO.0306-21.2021 (PMC8482853; doi:10.1523/ENEURO.0306-21.2021)
Supplement: Extended Data 1 — Code used to detect and analyze events in Igor Pro (coding-project) and for data analysis in R (PNA-KNDy). Download Extended Data 1, ZIP file. [file enu-eN-NWR-0306-21-s01.zip › extended-data-1/coding-project.zip!/coding-project-vbwregions/documentation/td analysis/ocvm information 20151015.doc]

OCVM Panel information

2015-10-15

The OCVM panel can be used to visualize the analysis of currents during voltage ramps. The main purpose is to obtain an estimate of the membrane potential without disturbing the intracellular milieu. This is accomplished when a sufficient number of A-type potassium channels are present during an on-cell (tight seal) recording. The channels can be manipulated by changing the command potential relative to the membrane potential of the cell (Vpatch = Vcell – Vpipette). Using the traditional whole-cell voltage conventions (Vpipette positive to ground), a strong depolarization of the patch (Vpipette = 200 mV) removes inactivation, a subsequent ramp (200 to -100 mV 50 msec) will activate the channels. Current flows according to the driving force. Assuming the pipette potassium concentration is similar to the intact cellular potassium concentration, the reversal potential for potassium should be approximately 0 mV. Thus, if sufficient current is flowing, the ramp potential (Vpipette) at which the activated potassium current reverses direction (Vpatch = 0mV) should be similar to the membrane potential of the cell (Vcell).

To analyze these data with the OCVM panel in the “td analysis” software, current and voltages traces obtained during the ramp are required. The linear region of the current evoked by the ramp at negative Vpatch potentials and prior to the activation of the potassium currents is linearly fit. This fit is subtracted from the entire ramp to subtract the leak (or passive) response of the patch.

The interface is accessed from the VC panel of the BlastPanel. First load the .dat file using the “Open PM bundle” button in the HEKA tab. Then click the VC panel, which should look like this:

/

Click the “OCVM panel” button (lower left). A new panel should open, looking something like this:

/

Click the labels popup menu (left center) and select the PGF name that matches the ramp data you wish to analyze. This will populate the listbox on the left. Once you click “Update”, ramps are automatically detected and the files are analyzed with the initial settings at the top. Of these, only Fit Offset and Fit Duration are relevant. Fit Offset is the value in seconds from the start of the ramp. Fit duration is the length of time in seconds for the linear fit. This is what it looks like after you click update.

/

Now click on the vertical grey bar on the listbox. This activates the listbox and allows you to use the up and down arrows to navigate the traces in the list box. Click the down arrow and the screen should look like this:

/

The blue highlight in the list box indicates which sweep is displayed. The black trace is the leak subtracted data. The red trace is the fit region. You can see that the red trace is not flat. If we decrease the fit offset and fit duration, we can make it flatter.

/

It’s important to examine all the traces/series to make sure these settings are optimal. This version of the software (OCVM v5_1, as of 2015-10-15), treats all the series with the same PGF label using the same analysis parameters which can cause problems if your recording is not stable. If your recording is unstable, it is likely you shouldn’t include the recording in your analysis. However, it is possible to ignore a trace by clicking the check box to the left of the listbox. This removes the sweep from the data table and the analysis graph (here shown on the left side of the OCVM panel). If you are changing parameters, it is useful to uncheck the “new display?” checkbox to avoid the creation of multiple graphs and tables.

The analysis graph shows the reversal potential as a time series using the time stamp PatchMaster applies to each sweep. Elapsed time from the beginning of the experiment is shown on the bottom axis. The top axis shows the absolute time. The y-axis is membrane potential.

If you have a double ramp to study the effect of an acutely applied agent such as GABA, and you check the OCVM-app check box, two additional data sets are created and displayed: the membrane potential obtained from the second ramp (in red) and the difference between the second ramp and the first ramp potential (in green). If you check “ramp 2” under the panel graph, the second ramp will also be displayed.

/
