# Supplementary material for: Prenatal Androgen Treatment Does Not Alter the Firing Activity of Hypothalamic Arcuate Kisspeptin Neurons in Female Mice
Source: eNeuro. 2021 Sep 29;8(5):ENEURO.0306-21.2021. doi: 10.1523/ENEURO.0306-21.2021 (PMC8482853; doi:10.1523/ENEURO.0306-21.2021)
Supplement: Extended Data 1 — Code used to detect and analyze events in Igor Pro (coding-project) and for data analysis in R (PNA-KNDy). Download Extended Data 1, ZIP file. [file enu-eN-NWR-0306-21-s01.zip › extended-data-1/coding-project.zip!/coding-project-vbwregions/documentation/td analysis/td_analysis manual 20160115.doc]

**Chapter 1: Installing TD Analysis**

Please note, filenames and folder names are in quotes to minimize confusion with long names.

You must use Igor Pro version 6.31. This version is available on the lab server.

Unzip the latest “td analysis20160115.zip” file. The date is appended to the file name at the time of compression and is usually less than six months old. If you have a version more than six months old please contact Tony at defazio@umich.edu.

Inside the TD analysis folder are two folders named “link to igor procs” and “link to user procs”. Make ALIASES (or shortcuts in the Windows world) of both of these.

Igor makes a folder called “Wavemetrics” that will be found in your “documents” folder (Mac) or “my documents” (PC). Inside the “Wavemetrics” folder, open the “Igor Pro 6 user files” folder. In there are folders called “Igor Procedures” and “User Procedures”. MOVE the aliases you created to the appropriate folder. Drag or copy the “link to igor procs” alias to the “Igor Procedures” folder; similarly, drag the “link to user procs” alias to the “User Procedures” folder.

Go back to the “TD analysis” folder and open the “link to igor procs” folder. COPY the file “IVtiming.txt”. MOVE the copy to “Igor procedures” folder inside the “Wavemetrics” folder (the one in your “documents” folder). RENAME copied file to remove the ‘copy’ and the space before copy. The name must be “IVtiming.txt”. Capitalization is important.

NOTE the “IGOR pro” folder in the applications folder contains folders with the same names as above BUT these should NOT be modified. Instead make sure all modifications are done in the “Wavemetrics” folder in the “documents”/”my documents” folder.

Occasionally, a release of the analysis routines will include multiple versions of a procedure file. In this case, please complain to Tony at defazio@umich.edu, and he will send you a cleaned-up version. In the meantime, you can survive this by “killing” the procedure files that are generating the errors. When Igor starts up, it compiles all procedures it finds in the “Igor Pro 6 User Files” folder and subfolders. If multiple versions exist, it throws an error dialog box that says “name already exists as a macro” or “name already exists as a function”. Simply click the “edit procedure” button. Close the procedure window. Igor throws a dialog that asks if you want to hide or kill the procedure window. Kill it without remorse. The alternative is to delete the offending file in the “td analysis” subfolders. Check the folders to see if there are more than one version, e.g. “tonys_procs v2.6.ipf” and “tonys_procs v2.7.ipf”. You can delete the versions with the smaller numbers and re-run Igor. This really should not happen anymore, but this situation can arise in older releases.

**Chapter 2: Brief Introduction to HEKA data structure**

PatchMaster files contain experiments, groups, series, sweeps, and traces in a tree-like structure compressed into a single “filename.dat” file. You should create a new PatchMaster bundled file (i.e., a new experiment) for EACH cell you record. An example tree as viewed in PatchMaster is shown in Figure 1. This .dat file contains 3 groups in experiment 1. These are the far left blue boxes, E1 1, E1 2, and E1 3.

At this time, the TD analysis routines do not display the first branch; the routines read all data, but assume everything is in E1, which it is if you start a new PM file for each cell. If you are running a particularly complex experimental design that requires multiple experiment branches of the data file, please email [tony.defazio@gmail.com](mailto:tony.defazio@gmail.com). TD analysis displays all the remaining branches of the tree (groups, series, sweeps, and traces). A single .dat file might contain multiple “groups”. In turn, each “group” contains a number of “series”, and each “series” consists of “sweeps”. Each “sweep” contains a “trace” of each channel recorded. A sweep is similar to an “episode” in pClamp and usually consists of sequence of voltage or current measurements obtained at a defined frequency (e.g. 10 kHz) Understanding how TD analysis will display these data can greatly speed up your ability to examine your files.

Figure 1. Reply window in PatchMaster illustrating file structure.

For data to display properly in TD analysis, it is important to set up your pulse generator file (PGF) correctly in Patchmaster. Within a PGF, the channel number you use to record a parameter (for example Im or Vm) becomes the trace number and this is carried over to TD analysis. If you have used multiple amplifier channels to record data, such as Im and Vm of cell1 and Im and Vm of cell 2, you will generate multiple traces (in this example traces 1-4). Note that PatchMaster will allow you to assign any acquired variable to any trace. To simplify your life, we really recommend that you design your .pgf patterns appropriately and in the same order for each PGF. An example pattern generator file is shown in . In this example, “Imon 1” is assigned to trace 1, “Vmon 1” to trace 2, “Imon 2” to trace 3, and “Vmon2” to trace 4.

Figure 2. Example pulse generator file for recording four traces (channels).

**Chapter 3: Importing your data from Patchmaster to Igor**

The software you installed in Chapter 1 makes available a series of macros in Igor Pro. To access these, start Igor and select “makeBlastPanel” from the macros menu. A panel appears with a series of tabs. The leftmost tab is HEKA. In this tab, there is a button labeled ‘Open PM bundled file’. Clicking on this opens a file dialog box. Navigate to the data file (i.e., “filename.dat”) that you wish to analyze.

The TD analysis routines create many new waves within Igor to facilitate analysis. It is thus necessary to keep the base filename as short as possible. This can be done with an 8-digit date code plus a letter to distinguish between cells on that day. For example, “20130311a” would indicate the first cell recorded on March 11, 2013. The TD analysis routines will issue a request to shorten your file name if you have exceeded 9 characters. You can put anything you like for the filename, if it is under 10 characters, but a little thought now will help you keep your data and your waves in Igor organized.

In the HEKA tab, the leftmost column of listboxes (red arrow in Error: Reference source not found) helps you navigate the file structure. By default, when your .dat file is loaded, all sweeps from the first group are displayed. If you click on a group (say “g1”), all series (and thus all sweeps and traces) in that group are displayed in “Analysis Graph 1”. If you want to view series 2 from group 3, first click on g3 then click on s2, Individual sweeps can be displayed by clicking on one of them.

The trace displayed in Analysis Graph 1 is determined by the selection in the traces listbox. Trace 1 is shown by default, but all of the individual traces for all the sweeps and series for a given group are available for selection in the column of listboxes labeled “AllPMwaves” (green arrow in Error: Reference source not found. Selecting one of these traces will cause it to be displayed in analysis graph 1. If you click the button “Import” near the bottom of the panel, the selected trace appears in the “imported waves” listbox to the right. Analysis requires the waves of interest to be listed in the “imported waves” listbox.

Figure 3. BlastPanel. Red arrow indicates column of list boxes to use for data navigation. The green arrow indicates the AllPMwaves column.

Labels: 2015-10-27 PGF names in PatchMaster and Labels in Igor

Some issues have arisen regarding how the "td_analysis" routines handle PGF names in Igor. This document is intended as guidance to getting the most out of your PGF name and facilitating future analysis sessions. The most recent versions of the "td_analysis" software take store the PGF name as a label for every trace during the import into Igor. These are accessible in the wave note (use "browse waves" to see the wave note in Igor). The analysis software can use these labels to sort waves for analysis. A prime example of this is the passive analysis: when you click this button, all waves with "passive" in the PGF name are analyzed. This allows you to use a PGF to monitor on-cell stability prior to whole-cell ("passive-fast") and another PGF to collect and average 16 sweeps during a recording (call this "passive"). Both sets of PGFs will be analyzed by the passive analysis routines, allowing you to visualize the progression of the passive properties of the entire experiment.

Therefore, I encourage you to use PGF naming conventions to distinguish between data series in PatchMaster that will be analyzed in different ways. Another example is OCVM. In this case there are several types of OCVM experiments: single ramps to monitor Vm over a long period of time (e.g. "OCVM-long"), double ramps to assess the effect of a neurotransmitter (e.g. "OCVM-double"). However, mild chaos will ensue if you use a passive protocol during an OCVM routine and call that PGF "OCVM-passive", since these traces won't have ramps for the OCVM analysis software.

The ability of the software to read the PGF name is new and can greatly facilitate analysis. If you want advice on naming conventions to optimize analysis, please feel free to talk to me. Furthermore, if you're having trouble with labels from old experiments, I can help you figure out how to make the best of it.

To view traces base on label, use the drop down list box at the bottom left hand corner of the Heka tab of the blastpanel (indicated by the yellow arrow in the screen grab below. You can select which trace number by checking the boxes.

**Chapter 4: Passive Analysis**

The purpose of a “Passive” PGF is to test the passive properties of a whole-cell voltage-clamp recording, including holding current, input resistance, capacitance, and series resistance.

“Passive” includes ten sweeps of -5mV hyperpolarizing steps, and the passive properties are calculated from the resulting the currents. For more information on how these calculations are made, please see Lesson 1 in the Moenter lab electrophysiology tutorial.

PGF

Below is an example of a “Passive” PGF in HEKA PatchMaster. In order to make the resulting data analyzable with the “makeBlastPanel” macro in Igor, there must be three segments of 20msec each: 1) holding potential, 2) step potential, 3) holding potential. Note that this PGF includes 10 sweeps so the average of the 10 resulting current traces can be calculated during analysis.

Ideally, “Passive” will be run periodically during a recording to monitor passive properties throughout the recording. In the example data shown in this chapter, “Passive” was run approximately every 30 seconds during the experiment.

Running Passive Analysis in Igor Pro

1) After opening the Patch Master-bundled file using the “makeBlastPanel” macro, run passive analysis by checking the box next to “Auto passive analysis” (red arrow).

Checking this box will automatically run the analysis, which performs the following tasks:

a. Error checking: Detects within the traces noise spikes and postsynaptic currents that will throw off the measurement of passive properties. Traces with these irregularities are rejected and noted in the command window.
b. Averages the 10 traces from each “Passive” series
c. Calculates passive properties [holding current, input resistance, capacitance, and series resistance (as explained above)] from each average
d. Plots the measured/calculated passive properties in a graph that looks like modern art:

The experiment was initiated at 10:59:10 AM, so the beginning of this graph includes points from some passive series run during set-up, before the experiment was initiated.

2) Select which properties to view by checking or unchecking the boxes color-coded for their corresponding traces (red circle).

The colors represent each property as follows:

Blue: holding current (HC)

Black: input resistance (Rin)

Green: capacitance (Cap)

Red: series resistance (Rs)*

*note that the top axis “Elapsed time” is linked to Rs, so it is only labeled when Rs is selected

Multiple properties can be displayed at the same time:

3) The axis ranges can be changed to view the data more clearly. For example, the view above excludes some Rin data points, so the Rin range can be expanded. To modify an axis, double-click one of the numbers labeling that axis to open the “Modify Axis” window with the “Axis Range” tab selected:

To change the range, type new values into the “Manual Min” and “Manual Max” fields. You can see these changes live in the background by checking the “Live Update” box is checked (red arrow):

To change a different axis, select a new axis from the “Axis” drop-down menu (red circle):

Axes:

left = holding current

bottom = absolute time

right = input resistance

rseries = series resistance

cap = capacitance

top = time relative to start of experiment (only visible with Rs)

To apply your changes in all axes, click “Do It” (red arrow)

[Note that the “Modify Axis” window is native to the Igor software and is not specific to the “makeBlastPanel” macro. Therefore, the modifications described in Step 3 are applicable to any graph created in Igor.]

The graph in Step 4 includes the changes applied on the axes of “Holding Current” and “Input Resistance.”

4) The values for any point in the graph can be viewed by hovering the mouse over that point (red arrows in the next two graphs). These values can be especially helpful in locating information in a table of the data (see step 5 below).

These values indicate that this point is the average input resistance in series 13 (s13) and that the point is at the time 11:01:41 with an input resistance of 614.4 MΩ.

These values indicate that this point is the average calculated holding current in series 13 (s13) and that the point is at the time 11:01:42 with a holding current of 48.604 pA.

5) To make a table of the data in the graph,
   a. make sure the graph window is active (explained in 5b), click the “Windows” menu at the top of the screen, and click “New Table…”

b. The “New Table” window will appear. Make sure the “Edit data columns only” radio button is selected. Check the box next to “From Top Graph” (red arrow). This will eliminate from the “Columns to Edit” list any options that are not part of the plots in the active graph. This is why making sure the graph window was active in step 5a was important.

c. Select any or all of the parameters you would like to view in the table. If a parameter is highlighted, it will appear next to “Edit” in the mini-command line:

Column options:

capa = capacitance

holdingc = holding current

Rinput = input resistance

Rseries = series resistance

Tstart = absolute time (time of the beginning of the first trace averaged for a given point)

Tstart_rel = time relative to start of experiment

To select all of the parameters, click the top parameter, hold the “Shift” button on your keyboard, and click the bottom parameter. This will highlight all of the parameters:

To select only some of the parameters, hold the “Ctrl” button (PC) or “⌘” button (Mac), and click/highlight the desired parameters.

d. Click the “Do It” button. A “Table” window will appear:

These values can be copied and pasted into an external spreadsheet, such as those in Microsoft Excel, for further analysis.

Should I explain how to draw a treatment bar, or is that still in development?

Update: On-cell subtraction for passive analysis 2013-09-30

Often it is not possible to completely compensate electrode capacitance when making voltage clamp recordings in brain slices. Generally, this is not a major inconvenience as long as raw and leak subtraction traces are acquired at sufficiently low gain to fully capture the large capacitive transients without clipping. However, a major concern is an accurate measure of series resistance. We measure this property from the peak of the capacitive transient during a 5 mV hyperpolarizing step. Since this transient includes both the electrode resistance and capacitance and the charging of the series resistance, input resistance, and the whole-cell capacitance, it is important to minimize the contribution of the pipette. In cases when the electrode capacitance cannot be fully compensated electronically, *post hoc* digital subtraction is necessary.

In this case, acquisition of the passive properties (response to a 5 mV step, use a step PGF [assuming HEKA PatchMaster] identical to the one you use in whole-cell) during the on-cell phase of whole-cell recording is extremely valuable. After baseline correction of the on-cell passive trace, subtraction from the whole-cell passive trace yields our best approximation of the series resistance and capacitance of recording configuration (pipette, seal, and cell). Since the properties of on-cell can be dynamic, it is important to capture the on-cell passive trace immediately prior to any degree of whole-cell access. Generally, this is evidenced by extremely fast transients (figure X.1c). I tend to acquire 2-5 on-cell passive series (16 sweeps each) as the seal stabilizes over the course of 1-2 minutes prior to going whole-cell.

In BlastPanel, load your .dat file using the “Open PM Bundled File” button. To select the on-cell passive series for subtraction during whole-cell passive properties analysis, click on the popup item titled “On-cell ave series” (Figure X.1). It is labeled “NONE” at the start of the analysis. Click it once to detect all passive series. Now when you click it, it shows the series number of each passive series. When you select a series, it is displayed in Analysis Window 1 (the blue one) as a red trace to distinguish it from other types of display. Now that you’ve selected the on-cell trace for subtraction, click on the Passive analysis check box. The selected trace will be subtracted from each passive series for capacitive and series resistance measurement. For reference, the average series are saved as filename_avl. The subtracted average series are saved as filename_avl_sub.

Figure X.1

**Chapter 5: Event detection**

***Principles of Event Detection***

The first principle of all data analysis is to never alter the original data. Although I have taken great pains to make it impossible for my routines to modify the raw data, you should always keep a back up of your original files before using these routines. Another critical concept is to establish reliable detection criteria early in the study and keep this fixed for all subsequent analysis. If you change parameters you can get quite different results, which necessitates re-analysis of all the raw data. Are there any exceptions to this? For example, sometimes if a recording is noisy but still has clear events it is necessary to change the threshold. Do you think this can ever be legit?

My approach to event detection is based on the first derivative and sequential heuristics. This means that an event is essentially a sudden change in signal with a predictable set of properties such as amplitude and area. The sudden change in signal shows up as a spike in the first derivative (Δy / Δt = (y_x+1_ - y_x_)/(t_x+1_-t_x_), where y is the sampled signal, x is the selected time point, and t is sample time). The predictable set of properties depends on the nature of your event. For example, action currents recorded extracellularly look much different from postsynaptic currents recorded in the whole-cell configuration.

The first thing the detection program wants to do is take the derivative. Unfortunately, there is always noise in the signal. Noise can look like an event to the detection software. Since the detection algorithm is going to search the first derivative for events, the ability to smooth both the raw data and the derivative (simple boxcar smoothing, deriv. pre-smooth and deriv. smooth, parameters respectively) have been built in to the analysis. Note the degree of smoothing depends on the type of data and sampling rate. In turn, the degree of smoothing will affect other detection parameters.

***BlastPanel event detection variables***

***Pre-smooth*** is a powerful tool for eliminating noise and speeding up the detection algorithm. This smoothing occurs before the derivative is taken and does not affect the wave containing the original signal (i.e., heavy pre-smoothing will not affect amplitude measurements). If there is an outrageously high number of potential events (>15,000), the detection routine will prompt you to make sure you want to proceed. Each potential event is checked with heuristics (if-then statements regarding amplitude and area). This is slow, so limiting the number of potential events without throwing away actual events is important. A potential problem can arise if you pre-smooth too much: you lose everything. The program lets you see the effects of pre-smooth in the derivative window after you click the graphic-user-interface (GUI) button. Test this by setting pre-smooth to “1” then click GUI. You should see the raw derivative of the original data in the second display window. Now change pre-smooth to 100 and see what you get.

***Derivative threshold*** is the main search parameter. It is marked in red in the list of detection variables and as a red line in analysis graph 2. Only events exceeding this threshold have a chance for automatic detection. Each time the derivative (after pre-smoothing and smoothing) crosses the derivative threshold, this is identified by the software as a “level crossing”. Each level crossing is a potential event. Both pre-smooth and smooth will affect the amplitude of the derivative, so you may need to adjust your threshold to compensate for heavy smoothing.

***Derivative smooth*** (note, not pre-smooth) occurs after the derivative of the original signal is obtained.

***Derivative max width*** and ***min width*** are further heuristics to eliminate noise. They refer to the half width of the spike in the derivative signal. For now, don’t mess with these settings.

***Window search*** determines the time the program will search for a peak in the raw data after the time the peak of a level crossing is detected in the derivative. A window search value of 2 msec will expect the peak of the event (not its derivative) to occur within 2 msec of the peak of the derivative. If you have slow rising events, you may need to increase this value. On the other hand, if the frequency of events is high enough to lead to superposition of events, you may need to shorten this window to properly detect superimposed events (otherwise, a burst of three events occurring within 2 msec will be counted as a single event).

***Peak sign*** determines the search for negative (-1) or positive (+1) events.

***Peak threshold*** is a heuristic, i.e. an if-then statement in the program compares the peak of the signal in the “Window search” with the baseline—if the amplitude does not exceed the peak threshold, the potential event is rejected. Peak threshold is marked in green in the detection variable panel and as a green line in analysis graph 1.

***Peak smoothing*** applies a boxcar smooth to the original data to smooth the peak. This can eliminate the effects of high frequency noise, but beware that setting this too high will diminish the amplitude. Depending on the noise levels, reasonable values for a sampling interval of 100µs are 3-10 points. During the initial validation of the detection settings, you should run several detection scenarios to convince yourself that the settings best reflect the data.

***Area threshold*** and ***Area window***: these parameters are used to calculate the area of the event (the area is calculated from the time of the derivative peak plus the area window) and accept or reject the event based on this threshold. This heuristic is meant to eliminate false events due to large, noisy spikes that sneak through the previous heuristics, but are too short to have adequate area like a postsynaptic current. These parameters depend on the type of event you are analyzing. The area of an action current is very small, a AMPA-mediated postsynaptic current larger and GABAA and NMDA mediated currents larger still. Careful thought about what you are analyzing can help you to set these parameters to reduce noise

***Baseline***

In order to measure the amplitude from the raw data, it is critical to determine the baseline current. These routines assume that the frequency of events is low enough to allow a baseline value to be measured prior to each event.

***Baseline duration*** and ***baseline offset***: offset is measured to the left of the derivative peak. Duration is taken backwards from the left of the offset, so you can have a short offset and long baseline if necessary. Baseline current is calculated from the original data (not the derivative) and is shown on the Analysis Graph 1 as a blue line in manual detection mode or after clicking the GUI button. Please see Figure XX. Offset should be relatively short (<=1-2 msec, even shorter for rapid events or high frequencies) since the derivative level crossing already precedes the peak. The baseline duration should be long enough to get a reliable mean from noisy data (5-10 msec should be sufficient under normal circumstances; high frequencies might require shorter baseline duration to avoid contamination of the baseline by the peak and/or tail of the preceding event).

***Trace duration***, ***trace offset***, and ***average cutoff***: These are parameters for display and for selecting events for averaging during the detection process (most useful for postsynaptic currents). The trace duration is the total duration of the averaged trace that will be displayed. Make this long enough to accommodate your events. Trace offset refers to the amount of time before the derivative peak included in the average. As written, offset is a component of duration so include the duration of offset you want in the trace duration. The software rejects events for averaging (but not for amplitude or interval) if a second event occurs within ”trace offset” ms of the derivative peak. Average cut-off is to remove really big events that might throw off the average. This parameter should be set very high. The original purpose was to exclude action currents during spontaneous PSC recordings.

***Import/export settings***: Once you have settings that work well, use the export settings button to save this somewhere safe. To load saved settings, click import settings and navigate to the file. I don’t recommend changing settings between files once you start your final analysis. Ideally, you should use the same settings for all analyses.

***Graphic-user interface***

The graphic-user-interface (GUI) button (top middle) brings up graphs showing the original data (analysis graph 1) and the derivative (analysis graph 2) with lines indicating settings for derivative threshold (red, graph 2), peak threshold (green graph 1) and baseline (blue graph 1). The duration of the display in seconds is set by xmin and xmax in the top left corner. Note your trace duration does not set these values automatically so you may need to adjust them to see an entire trace!

After you’ve clicked GUI, the lines for derivative threshold, peak threshold and baseline will live update as you change the parameters.

You can navigate in the analysis graphs using the normal Igor tools (expand, horizontal expand, etc). The align button can be used to align the X axis scales of the derivative with that of the original data after you are happy with the scale in analysis graph 1. If you change the scale on the raw data to zoom in on a single spike, then click align, you will see that event’s derivative. To keep this zoom, click on the “get” button next to xmin and xmax.

***Detecting events, plotting results and rejecting bad events***

After setting your parameters you are ready to detect events. The detect button initiates the process. If >15,000 level crossings are detected with your chosen parameters, a dialog box will appear asking if you wish to continue with detection. Enter 1 for yes and 0 for no. If there are too many events (say 50,000 in two minutes), you may want to increase your pre-derivative smoothing. If there are too few, you may need to decrease your pre-derivative smoothing.

### Detection modes

#### **Auto detection**

If the auto detection box is checked, the software will proceed through the entire data set, automatically detecting events. Really, this is what you want to do! Manual detect is not recommended.

***CEREBRO: Event navigation for deleting events***

CEREBRO plots an array of events allowing for quick screening for elimination of bad events. Make sure your wave is selected in the “imported waves” box. Select “events” in the analysis list box and “CEREBRO” in the plot types list box and click plot. By default CEREBRO will plot a 5x5 array of the first 25 events; this can be adjusted in the control panel at the bottom of the page. You can select raw data only, derivative only or both to be displayed for each event. The dashed line in the middle of the panel identifies the event under consideration in that panel (depending on event frequency, other events may show up in the panel). Click anywhere in the panel of an event that you wish to delete. CAN”T go further on CEREBRO as newest version doesn’t run on my computer and I can’t see the control boxes, sigh. Note to self, no updates before RAD leaves town!

***Wave navigator: event navigation for adding and deleting events***

To navigate through the detected events, select “events” in the analysis list box, and “wave intrinsic” in the plot types list box, and click “plot”. This will open the wave navigator. This panel has two graphs separated by a control bar and IgorPro graph information on the bottom. The top graph plots raw data; events are marked by red circles in the raw data display. These show the measured amplitude based on the peak smoothing parameter. The derivative peak is shown below with a blue dot. The bottom trace plots the derivative; level crossings are marked with blue circles. The baseline (blue, top graph), peak threshold (green, top graph) and derivative threshold (red, bottom graph) lines are also visible on this panel.

##### Event navigator control bar

• previous and next navigate between detected events.

• A particular event number can also be entered. Entering 0 will navigate to the beginning of the data.

• Delete and add are used to remove or include events in the final analysis.

• Previous level and next level navigate between level crossings (derivative greater than derivative threshold)

• A particular level number can also be entered

• Backward and Forward navigate along the entire trace in increments of the trace duration

• To navigate to the beginning of the data set, enter 0 into event number

##### Deleting events

Clicking the delete button will automatically delete the active event at left side of the screen. Probability distributions are updated automatically

##### Adding events

Select cursor A from the bottom left of the wave navigator panel. Drag the cursor to the top graph and place it near the peak of the event to be added. Click the add button. The event detection dialog box will appear and ask if you want to add this event to the database. Click continue and a green indicator dot will appear over the event and the derivative. If green indicator dots don't appear (or they are not where you want them), reposition the cursor and click Add again until you are happy with the position of the green dot. Click Add one more time and now enter a 1 instead of the default zero. The green dot will turn red and be added to the database and all distributions (but not averaged traces at this time) will be updated.

### Plotting data after detection

Select the imported wave (it should be highlighted); select the parameter you want to plot (e.g., interval) in the analysis for display list box; finally, select the plot type (e.g., probability distribution) and click plot. X-Y and histogram plots are not yet available. Not all parameters are measured at this time (e.g. fwhm, 10to90 decay, tau, risetime).

##### Plot types

Probability distributions plot the parameter on the x-axis (say amplitude) and the cumulative probability is plotted on the y-axis. This is simply the data sorted by parameter then normalized by the number of events.

Need to add info on what parameters autoupdate upon event addition and deletion

**Chapter 6: Data analysis: Voltage Clamp**

One thing that I can’t remember if I mentioned earlier is the ability to average data before analysis. Chu does a lot of stuff where he is measuring small things and needs to do this. Likewise on the ramp studies we are averaging 3-5 runs to reduce noise, as we did for the series 2.

Need some lead in to what this is!

1. In the HEKA tab, open 26Nov07_2.dat by clicking the “Open PM bundled file” button. All data will import automatically for VC and CC analysis. All data traces will appear in the blue box and all command protocols in the yellow box. This is an example from a specific study-it should be introduced in a way that makes this clear. Maybe different margins? This is indented but that is really all that suggests this is different.

I’m thinking of my physics book, which had clearly identified sample problems

2. Select a series to analyze. Let’s try series 3. Click “s3” in the Series list box (indicated by the red arrow above). Now you should see the data and voltage protocol only from this series in analysis graph one?. The analysis will operate on whatever data is selected on this page displayed in analysis graph 1?. It is possible to select a group, series, sweep or trace for analysis.

3. Let’s make an IV curve. Click on the VC tab (green arrow in figure xx above). Now you should see the VC analysis tools (Figure yy below).

4. Click on the “Cursors” button to display the vertical lines in “Analysis graph 1”. The red cursors are for baseline adjustment, the blue for series resistance estimation (if you have a test pulse in your protocol, which we highly recommend but isn’t in this example-should we change this to an example with SR adjustment?), and the green lines are for the data analysis range. Slide the baseline and series resistance (red and blue) lines to the far left using the sliders in the VC tab (indicated by red arrow on Figure zz –note you won’t see this arrow on your screen). Use the sliders to move the green cursors to the region of interest. Your screen should look like this Figure aa

4alt. Alternatively, you can load cursor settings from the list box. Scroll down to “minimum” DOES NOT EXIST haven’t had time to start up just reading for clarity(it should right below final2, let me know if it is still missing STILL MISSING BUT WAS ABLE TO ADD using save button), and select it. Note that once you have settings you really like, you can save them by clicking the “save” button for future use.

Now click the “measure” button. There are four variants: “mean” measures mean between the cursors, “min” takes the minimum, “max” takes the maximum, and “smart” uses a simple algorithm that guesses what you are looking for (it takes the larger of the minimum and the maximum for each sweep, useful for IVs that cross zero and you can’t use the mean).

Smoothing invokes a boxcar average of the data between the green? cursors before the measurement. Keep track of what you do so you can be consistent as smoothing here alters neither the raw nor the imported data; the smoothing occurs in a copy of the relevant data, which is subsequently deleted. Series R applies a series resistance correction to the voltage based on the absolute current and Ohm’s law. The Series R value is either calculated from the trace itself (if enabled with the blue cursors) or using a value that you enter in MΩ (enter 10 for 10 MΩ). Enter 0 if you don’t want any correction. Will it be possible to select multiple data analysis parameters (mean, max)? Or will you just re run if you want multiple parameters reported? For max/min, is it possible to make it average for a window of xx points around a peak so you don’t just get a noise spike?

5. Click measure or plot? and the following graph will appear.

6. Currently the data are stored in “IVcorrected” and “IVxcorrected”. If you want to save these waves, you need to rename them. The next version will do this for you.
7. The software will report (in the history window) the reversal potential if the trace crosses zero. What about cases in which Vrev is where two lines cross (eg. Before and after drug x that modifies current) like we did in GABA paper?

What about the plot button?Shows a different graph that needs a bit o explaining. Got some splainin to do?

Also if imported waves box isn’t going to be used maybe kill it and clear imported waves button?

For min and max, is this a single point or is an average of a range? I’d prefer the latter for something like an IA measurement. One point is just so one pointy. OK see current comment above, one thing is consistent!

RAMPS

1. Now click on series 2 in the HEKA tab. The raw data appear in the top graph and the voltage ramp created from the information in the protocol is shown in the bottom graph. Click on the VC tab to analyze the data that appear in these graphs

2. There is some weirdness in the HEKA protocols, which I am resolving with their programmers (see the extra stuff at the end of the voltage protocol). Also, if the protocols change (i.e. added number of sequences), my routines will fail to create a waveform from the protocol. Please email if you encounter this bug: [tony.defazio@gmail.com](mailto:tony.defazio@gmail.com).
3. Once in the VC panel, click on the cursors button and move the red and blue cursors to the far left (or select the “minimum” cursor setting from above). Move the green cursors to the beginning and end of the ramp as below. (FYI the minimum I created worked).

4. Click on the “plot” button to create a plot of the membrane current as a function of the voltage ramp. All the sweeps in the blue graph (analysis graph1) are plotted in the new graph as a function of the voltage in Analysis Graph 2. Sweet. Measure doesn’t seem to work with this-should it- everything is -65.57 V Right now, the Measure button only operates on Analysis Graph 1 and it plots vs. data in Analysis Graph 2. I’m working on setting it up to do a time course analysis. These each have unique names so you don’t have to rename them as in the IV data.
